# Synthesis and Properties of 6-Aryl-4-azidocinnolines and 6-Aryl-4-(1,2,3-1*H*-triazol-1-yl)cinnolines

**DOI:** 10.3390/molecules24132386

**Published:** 2019-06-27

**Authors:** Natalia A. Danilkina, Nina S. Bukhtiiarova, Anastasia I. Govdi, Anna A. Vasileva, Andrey M. Rumyantsev, Artemii A. Volkov, Nikita I. Sharaev, Alexey V. Povolotskiy, Irina A. Boyarskaya, Ilya V. Kornyakov, Polina V. Tokareva, Irina A. Balova

**Affiliations:** 1 Institute of Chemistry, Saint Petersburg State University (SPSU), Universitetskaya nab. 7/9, Saint Petersburg 199034, Russia; 2 Department of Genetics and Biotechnology, Saint Petersburg State University (SPSU), Universitetskaya nab. 7/9, Saint Petersburg 199034, Russia; 3 Laboratory of Nature-Inspired Technologies and Environmental Safety of the Arctic, Kola Science Centre, Russian Academy of Sciences, Fersmana 14, Apatity 184209, Russia

**Keywords:** azides, cinnolines, triazoles, CuAAC, alkynes, cycloalkynes, Richter cyclization, Suzuki coupling, fluorescence, cytotoxicity

## Abstract

An efficient approach towards the synthesis of 6-aryl-4-azidocinnolines was developed with the aim of exploring the photophysical properties of 6-aryl-4-azidocinnolines and their click reaction products with alkynes, 6-aryl-4-(1,2,3-1*H*-triazol-1-yl)cinnolines. The synthetic route is based on the Richter-type cyclization of 2-ethynyl-4-aryltriazenes with the formation of 4-bromo-6-arylcinnolines and nucleophilic substitution of a bromine atom with an azide functional group. The developed synthetic approach is tolerant to variations of functional groups on the aryl moiety. The resulting azidocinnolines were found to be reactive in both CuAAC with terminal alkynes and SPAAC with diazacyclononyne, yielding 4-triazolylcinnolines. It was found that 4-azido-6-arylcinnolines possess weak fluorescent properties, while conversion of the azido function into a triazole ring led to complete fluorescence quenching. The lack of fluorescence in triazoles could be explained by the non-planar structure of triazolylcinnolines and a possible photoinduced electron transfer (PET) mechanism. Among the series of 4-triazolylcinnoline derivatives a compound bearing hydroxyalkyl substituent at triazole ring was found to be cytotoxic to HeLa cells.

## 1. Introduction

**Figure 1:**
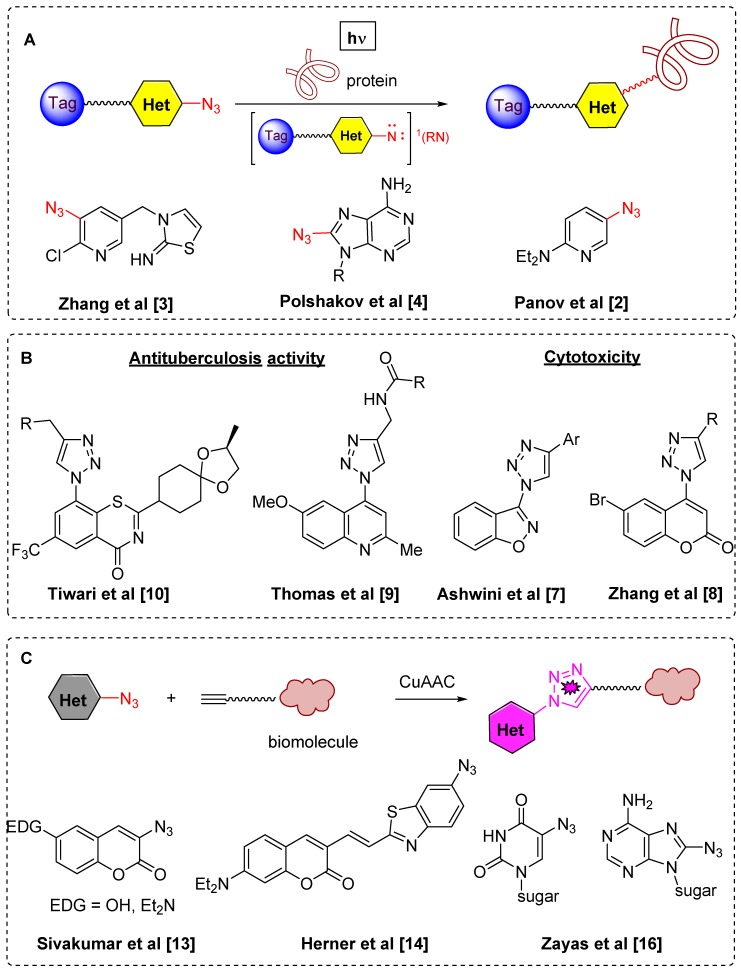
Recent areas of azidoheterocycles and triazolylheterocycles application–photoaffinity labeling (**A**); biologically-active compounds (**B**); fluorogenic azides (**C**).

During last decade azido-substituted heterocycles have been broadly studied in several areas. The first one is photoaffinity labeling [1] which is an important biological tool for the investigation of different biological processes by specific modification of proteins using their interaction with singlet nitrenes generated from azidoheterocyles under UV irradiation (Figure 1A) [2,3,4]. The second approach involves use of azidoheterocycles in the synthesis of triazolylheterocycles, mainly by Cu-catalyzed alkyne-azide cycloaddition (CuAAC) [5,6]. This methodology allowed a great variety of biologically active triazolyl substituted heterocycles to be synthesized and tested in numerous biological assays (Figure 1B) [7,8,9,10]. The third main field where azidoheterocycles are in great demand is as azide-based fluorophore tags for biological imaging (Figure 1C) [11,12,13,14,15,16].

**Figure 2:**
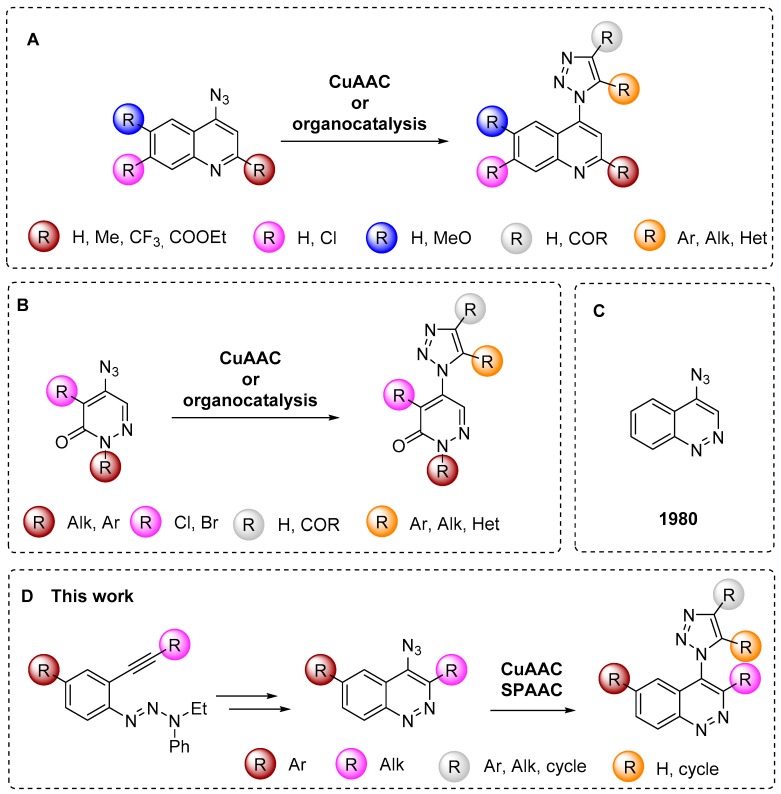
Known examples of azidoquinolines, azidopyridazines and the corresponding triazoles compared to azidocinnoline derivatives (**A**–**D**).

Among azidoheterocycles, azidoquinolines (Figure 2A) [17,18,19,20,21,22,23] and azidopyridazines (Figure 2B) [24,25,26,27,28] are well known, however only one known example of an azidocinnloline has been reported to date (Figure 2C) [29], while triazolylcinnolines still remain unknown. Taking into account that the cinnoline core can be found both in biologically active compounds with different types of activities [30,31,32,33,34] and in fluorescent materials [35,36,37,38,39,40], the development of a synthetic route towards azidocinnolines and triazolylcinnolines could lead to new biologically active molecules and interesting fluorescent probes.

## 2. Results and Discussion

### 2.1. Synthesis of 6-aryl-4-azidocinnolines

Several routes have been established for introduction of an azido group into a heterocyclic ring [41]. The most common techniques are azidodediazoniation of arenediazonium salts, synthesis of azides from the corresponding heteroarylboronic acids and nucleophilic substitution of activated halogens. In most cases involving 4-azidoquinolines nucleophilic substitution of a chlorine atom at the C4 position of a quinoline ring has been employed [42,43,44,45]. A few examples are known of bromine substitution [46,47] along with azidodediazoniation [48] and conversion of the corresponding quinolones to azidoquinolines employing diphenylphospharylazide [49].

The synthetic routes towards cinnolines include different cyclization techniques and named reactions [33,50,51]. One of them, which allows 4-halocinnolines to be obtained in high yields, is the Richter-type cyclization [52] of *ortho*-ethynylarenediazonium salts [53] and *ortho*-ethynylaryltriazenes [54,55,56]. The halogen atom at C4 position can be substituted with various nucleophilic reagents, i.e., water [53], alcoholates [57,58,59], sulfides [56,58,60] and amines [58,61,62,63,64]. The only mentioned example of a 4-azidocinnoline molecule was also obtained from the corresponding chlorocinnoline by nucleophilic substitution with sodium azide in ethanol [29].

**Scheme 1:**
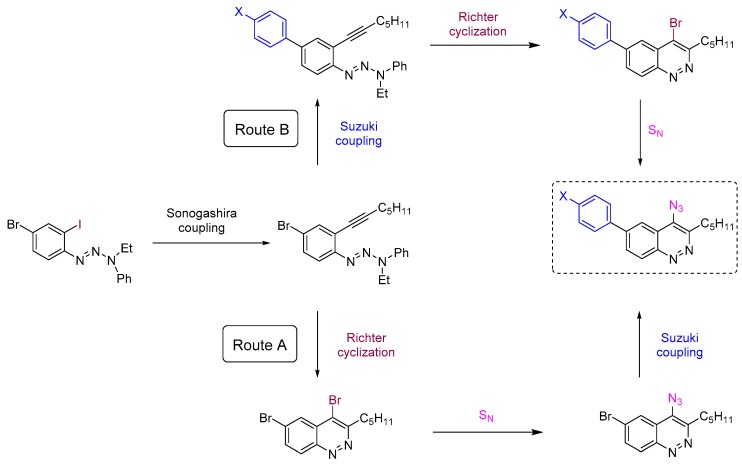
Proposed synthetic “Routes A“ and “B” towards 6-aryl-4-azidicinnolines.

To reach the target 4-azido-6-arylcinnolines compounds we decided to use 4-bromocinnolines, which are synthetically accessible by the Richter cyclization in higher yields compared to corresponding chloro derivatives [54], while the bromine atom at the C4 position should be still reactive towards nucleophilic substitution. Firstly, two synthetic routes (A and B) have been proposed for target molecules (Scheme 1).

The first step for both routes is the chemoselective Sonogashira coupling of the C-I group of 4-bromo-2-iodophenyltriazene with hept-1-yne (Scheme 1), which has been used previously for the synthesis of poly(arylene ethynylene)s [40].

**Scheme 2:**
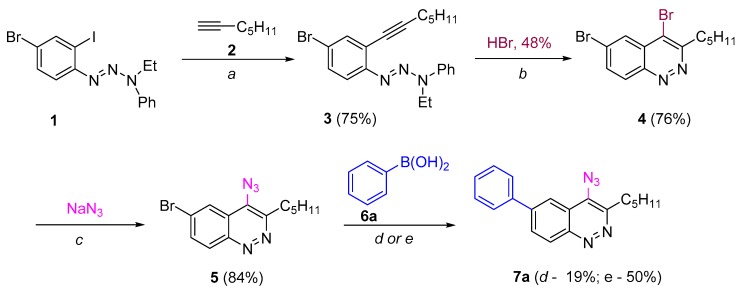
Synthesis 6-aryl-4-azidocinnolines through “Route A”. Reagents and conditions: (**a**) Pd(PPh_3_)_4_, CuI, Et_3_N, THF, 40 °C, 5 h; (**b**) HBr (20 eqiuv), acetone, 0.01 M, rt, 10 min; (**c**) NaN_3_ (5 equiv), 50 °C, 24 h; (**d**, Method A) − Pd(PPh_3_)_4_, Na_2_CO_3_, toluene/dioxane/H_2_O, 80 °C, 1 h, (**e**, Method B) Pd(PPh_3_)_4_, K_3_PO_4_, dioxane, 80 °C, 1 h.

“Route A” seems to be more divergent and rational than “Route B”, because it allows for variations of the aromatic substituent during the last synthetic step. Therefore we chose “Route A” to start with. The Richter-type cyclization followed by the regioselective substitution of bromine atom with sodium azide in absolute DMF at the activated C4 position proceeded smoothly and did not affect the bromine atom at C6. Hence 4-azido-6-bromocinnoline **5** was obtained in good yield (Scheme 2). Next we tried to carry out the Suzuki coupling. Despite the fact that this transformation of substrates bearing aromatic azido groups is known, yields usually are low, because the whole process is accompanied by several side reactions that are common for both Suzuki coupling (homocoupling, reductive dehalogenation) and for azides (denitrogenative decomposition through nitrene intermediates and through the Staudinger reaction if phosphine ligands are present in the reaction mixture) [65]. Two types of conditions tested differed in the base (Na_2_CO_3_ or K_3_PO_4_) and solvents (toluene/dioxane/H_2_O or only dioxane, respectively) used (Scheme 2). The formation of the desired 4-azido-6-phenylcinnoline **7a** as the main product was observed in both cases. However the isolated yields of the product **7a** were found to be between low (conditions *d*, Scheme 2) to moderate (conditions *e*, Scheme 2). Moreover the Suzuki coupling proceeded with the formation of many byproducts that required time-consuming purification of the target azide by column chromatography. Therefore we turned to “Route B”.

**Scheme 3:**
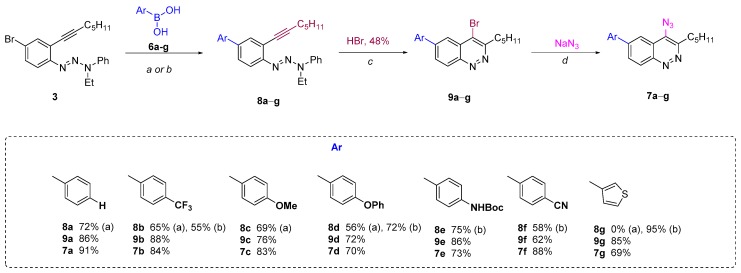
Synthesis of 6-aryl-4-azidicinnolines **7a−g** through “Route B”. Reagents and conditions: (**a**, Method A) Pd(PPh_3_)_4_, Na_2_CO_3_, toluene/dioxane/H_2_O, 100 °C, 24 h; (**b**, Method B) Pd(PPh_3_)_4_, K_3_PO_4_, dioxane, 100 °C, 2−5 h; (**c**) HBr (20 eqiuv), acetone, 0.01M, rt, 10 min; (**d**) NaN_3_ (5 equiv), 50 °C, 24 h.

The Suzuki coupling of aromatic halotriazenes has been employed recently in the initial steps of the synthesis of hexadehydrotribenzo[12]annulene [66]. Similar conditions (Na_2_CO_3_, toluene/dioxane/H_2_O, 100 °C) for our substrates gave 4-phenyltriazene **8a** in 72% yield. Applying next the Richter-type cyclization and nucleophilic substitution of bromine with sodium azide under the same conditions as for “Route A” enabled us to produce the final 4-azido-6-phenylcinnoline **7a** in better overall yield (42%, “Route B”) (Scheme 3) compared to “Route A” (24%) (Scheme 2). Same routes?

The scope of “Route B” was then checked with different aryl- and heteroarylboronic acids **6b**–**g**. “Conditions *a*” worked well for the CF_3_ (**b**), MeO (**c**) and PhO (**d**) series. However, these Suzuki coupling conditions failed when applied to other boronic acids bearing NHBoc (**6e**), CN (**6f**) and 3-thienyl (**6g**) groups. Thus, either complete or partial decomposition of the starting triazenes occurred when a mixture of organic solvent with water in the presence of sodium carbonate as a base was used. Excluding water from the reaction mixture and changing the base to potassium phosphate allowed all three triazenes **8e**–**g** to be obtained in high yields. The Richter cyclization and the subsequent nucleophilic substitution of bromine by an azido group for all compounds proceeded smoothly under the same conditions as for the unsubstituted phenyltriazene **8a**, providing a series of key 6-aryl(heteroaryl)-4-azidocinnolines **7** in high yields (Scheme 3).

### 2.2. Study of 6-aryl-4-azidocinnoline’s Reactivity in the Sythesis of 6-aryl-4-triazolylcinnolines

**Scheme 4:**
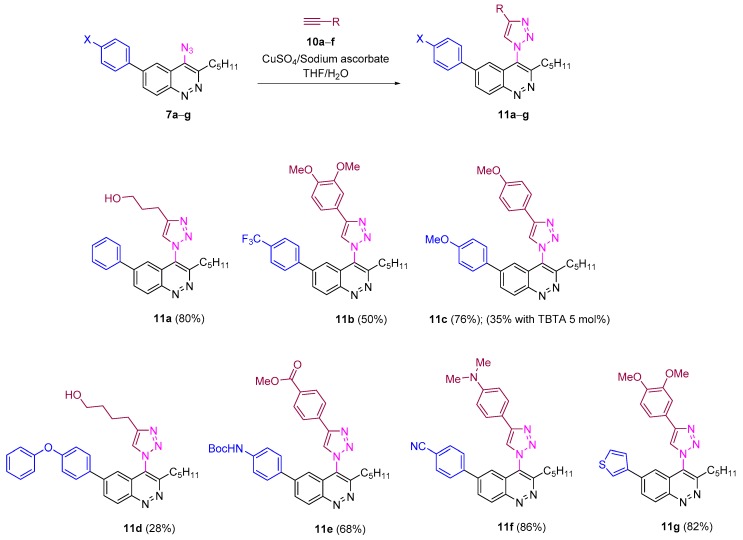
Reactivity of 4-azidocinnolines **7a−g** in CuAAC.

All azidocinnolines were found to be stable crystalline compounds when stored at −18 °C and slowly decomposed in solution. Thus complete decomposition of azidocinnoline **7a** in acetone-*d_6_* was detected after a week. Despite this fact, all azides were stable under conditions used for CuAAC. Thus carrying out CuAAC of azidocinnolines **7a**–**g** both with terminal aromatic alkynes bearing EWG, EDG and aliphatic alkynes in the mixture of THF/H_2_O using the copper (II) sulfate/sodium ascorbate catalytic system afforded the corresponding 4-triazolylcinnolines mostly in good yields (Scheme 4).

One modification of the well-studied CuAAC is to carry out the reaction in the presence of tris(triazolyl) ligands, which improves the yields, shortens the reaction time and improves the synthetic accessibility of triazole derivatives that are not available when the common Cu(II)/ascorbate catalytic system is used [67]. Surprisingly, when the CuAAC of azidocinnoline **7c** with 3,4-dimethoxyphenylacetylene was run in the presence of the TBTA ligand (tris[(1-benzyl-*1H*-1,2,3-triazol-4-yl)methyl]amine), the reaction proceeded very slowly and full conversion was not achieved even after two days. Therefore triazolylcinnoline **11c** was isolated in only 35 % yield (the yield of **11c** without TBTA ligand was 76%).

**Scheme 5:**
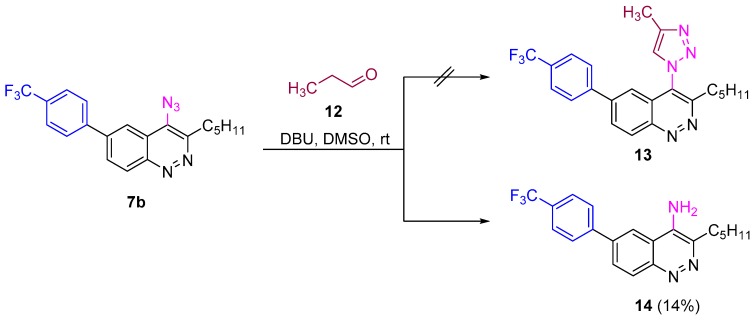
Reactivity of azidocinnoline **7b** in enol-mediated organocatalytic triazole synthesis.

Next we checked the reactivity of azidocinnolines synthesized in reactions other than CuAAC used for the synthesis of 1*H-*1,2,3-triazole derivatives, like the enol-mediated organocatalytic synthesis of triazoles and strain-promoted alkyne-azide cycloaddition (SPAAC). Unfortunately, enol-mediated reaction [68] of azide **7b** with propionic aldehyde catalysed by 1,8-diazabicyclo-[5.4.0]undec-7-ene (DBU) did not go as expected. The only isolated product was 4-aminocinnoline, which was presumably formed from the corresponding nitrene as an intermediate of azide decomposition (Scheme 5).

**Scheme 6:**
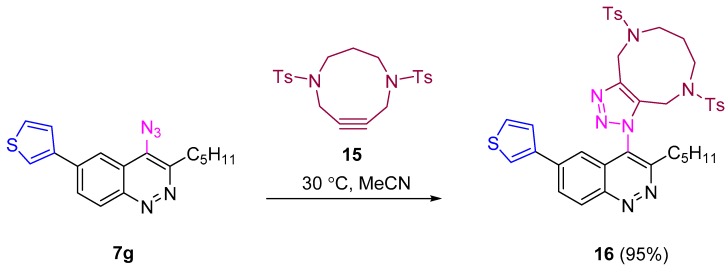
Reactivity of 4-azidocinnoline **7g** in SPAAC.

SPAAC of thienylazidocinnoline **7g** with tosylated diazacyclononyne [69] proceeded smoothly at 30 °C and afforded corresponding triazole in excellent yield (95%, Scheme 6). Thus synthesized 6-aryl-4-azidocinnolines were found to be suitable substrates for the synthesis of 6-aryl-4-triazolylcinnolines through both CuAAC and SPAAC.

### 2.3. Photophysical Properties of 6-aryl-4-azidocinnolines and 6-aryl-4-triazolylcinnolines

Having both azidocinnolines and triazolylcinnolines in hand, we next studied their photophysical properties. Fluorogenic azides represent a class of fluorescent dyes which are only weakly fluorescent without conversion into triazoles. However, when a triazole moiety is introduced into the molecule, it imparts fluorescent properties to the entire molecular scaffold. Fluorogenic azides are of special interest for bioimaging, because they allow avoiding a series of steps to wash the excess of dye from the labeled materials and thus they eliminate the problem of background fluorescence. Syntheses and applications of various fluorogenic heterocyclic azides have been reported recently and discussed in the Introduction section [11] (Figure 1). Taking into account that the aryl substituent, azide group and azo-group of a cinnoline core are conjugated, we decided to investigate whether 6-aryl-4-azidocinnolines could be used as fluorogenic azide dyes.

**Figure 3:**
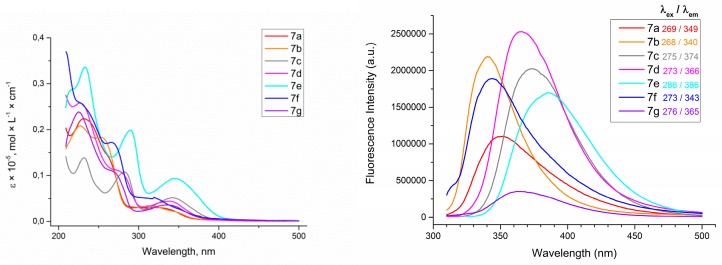
Absorption spectra of 4-azidocinnolines **7a−g** solutions in THF, C = 1 × 10^−5^ mol/L (left) and emission spectra of 4-azidocinnolines **7a−g** solutions in THF, C = 0.33 × 10^−5^ mol/L (right).

Firstly, the absorption and emission spectra of azidocinnoline solutions in THF were measured (Figure 3). The obtained data revealed that both EWG and EDG groups at the *para*-position of an aryl ring attached to C6 atom provided an increase in fluorescence brightness. To quantify the observed fluorescence, the absolute quantum yields of fluorescence (QY) of azidocinnolines **7** were measured (Table 1).

**Figure 4:**
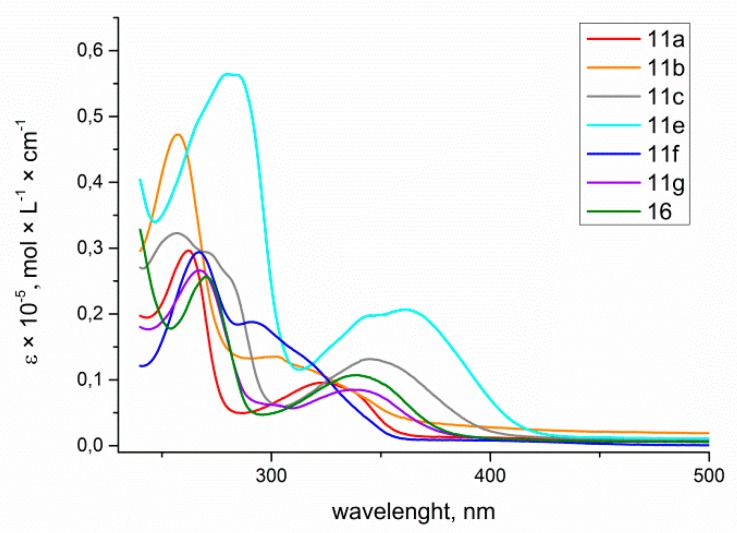
Absorption spectra of 4-triazolylcinnolines **11a−c, e−g, 16** solutions in THF, C = 1 × 10^−5^ mol/L.

**Figure 5:**
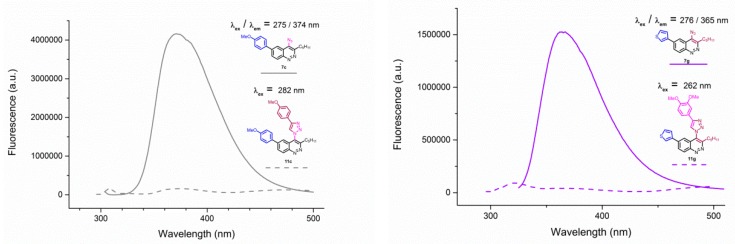
Emission spectra of 4-azidocinnolines/4-triazolylcinnolnes **7c/11c** (left) and **7g/11g** (right) solutions in THF, C = 1 × 10^−5^ mol/L.

The obtained data revealed that all 6-aryl-4-azidocinnolines **7** possess weak fluorescence with quantum yield values of less than 1%, with the highest QY being observed for 4-azido-6-(4-methoxyphenyl)cinnoline. Hoping to observe fluorescent properties in triazole derivatives **11, 16** the absorption and emission spectra for the series of triazolylcinnolines **11a**−**c,e**−**g**, **16** were measured (Figure 4 and Figure 5).

**Figure A2:**
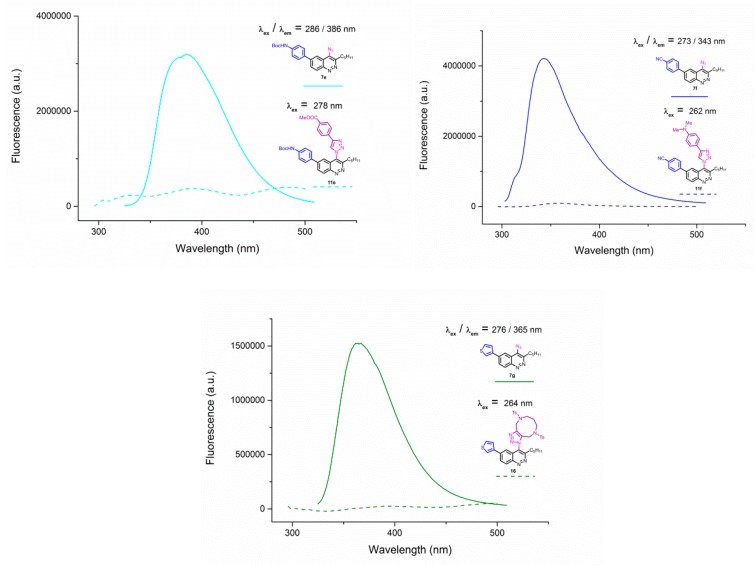
Emission spectra of 4-azidocinnolines/4-triazolylcinnolnes **7a/11a; 7b/11b; 7e/11e; 7f/11f; 7g/16** solutions in THF, C = 1 × 10^−5^ mol/L.

Unfortunately, all triazoles were found to be devoid of any fluorescence. Emission spectra of azide/triazole pairs with MeO group **7c**/**11c** and 3-thienyl group **7f**/**11f** are presented on Figure 5 (for the spectra of the whole series see Appendix A–Figure A1 and Figure A2).

**Figure 6:**
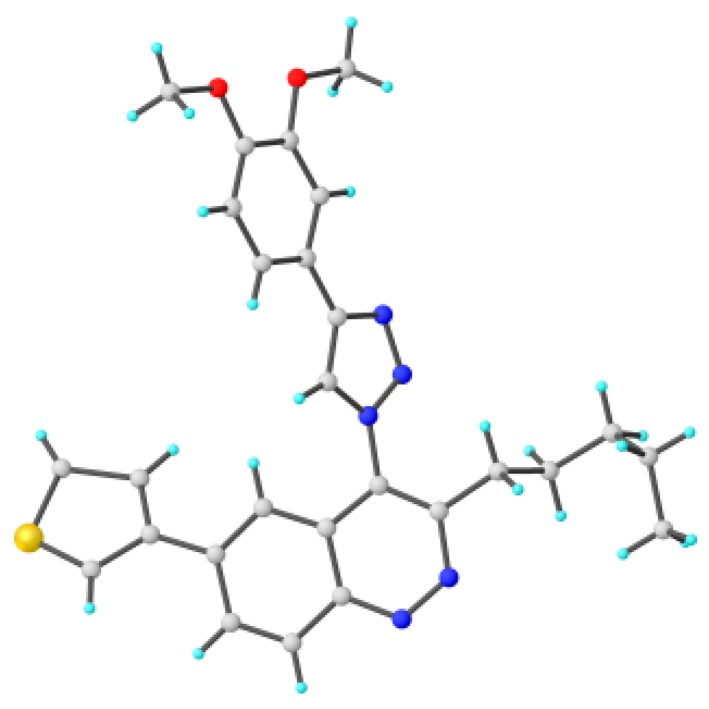
Molecular structures of triazolylcinnoline **11g** obtained from X-Ray analysis.

To explain the observed loss of fluorescence for triazoles, quantum chemical calculations for S^0^ states of the azide/triazoles pairs **7c**/**11c** and **7f**/**11f** were carried out. Geometry optimization was achieved using DFT calculations (B3LYP 6-311 + G(2d,2p)). The obtained data revealed that triazoles **11c** and **11f** are non-planar molecules: the triazole rings of both compounds lay out of the corresponding cinnoline plane (Table 2). X-Ray studies confirmed the non-planar geometry of triazole **11f** in the solid state (Figure 6). The dihedral angle between the triazole and cinnoline rings was found to be 64.8°. Therefore, despite the extension of the conjugated chain, the non-planar geometry of 4-triazolylcinnolines could be one of reasons for the observed absence of fluorescence.

Analysis of the frontier molecular orbitals of triazolylcinnolines **11c** and **11f** showed that the HOMO is associated with the donor part of the molecule (triazole) whereas the LUMO is concentrated in the acceptor cinnoline part. On the other hand the frontier molecular orbitals of azidocinnolines **7c** and **7f** are evenly distributed over the entire molecule (Table 2).

Therefore, another reason for the lack of fluorescence of triazolylcinnolines compared to azidocinnolines could be intramolecular photoinduced electron transfer (PET) that is known to be responsible for fluorescence quenching [70]. For example, 1,4-bis(2-hydrohyphenyl)-1,2,3-triazole with a similar frontier molecular orbital separation between both 2-hydrohyphenyl substituents exhibited very weak fluorescence [71].

### 2.4. Biological Studies of 6-aryl-4-triazolylcinnolines

Next we turned to biological activity screening tests. Both the triazole fragment and cinnoline moiety can be found in variety of compounds with antibacterial, antifungal and anticancer properties. Therefore we studied the antibacterial and antifungal activities of triazoles **11a,c,e−g, 16** against *Escherichia coli* and *Saccharomyces cerevisiae*, respectively. The cytotoxicity of triazolylcinnolines and their activity as DNA-cleavage agents were also tested.

**Figure 7:**
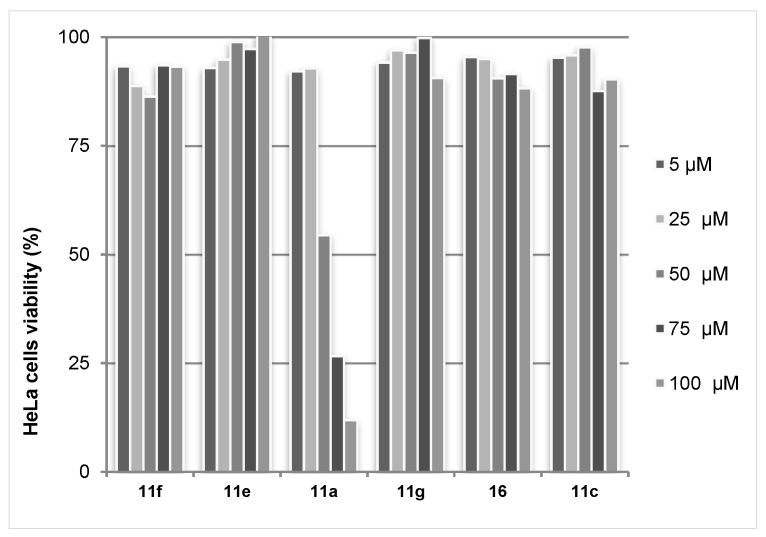
MTT test results with HeLa cell line for triazolylcinnolines **11,16**.

The obtained data revealed that the studied triazolylcinnolines **11a,c,e**−**g, 16** are inactive towards the Gram-negative bacterium *Escherichia coli* and fungus *Saccharomyces cerevisiae*. On the other hand the MTT test for screening of cytotoxicity allowed identifying triazolylcinnoline **11a** as being active. Despite the fact that other triazolylcinnolines did not display reduced HeLA cell viability, the IC_50_ for compound **11a** bearing hydroxyalkyl substituent was found to be 56.5 μM (Figure 7). Triazolylcinnoline **11a** possess a flat heteroaromatic moiety−6-arylcinnoline, which might have DNA intercalating activity. Therefore we tested the ability of triazolylcinnolines to cleave DNA. None of the compounds affected the DNA plasmid pBR322, indicating a different mechanism of cytotoxicity.

## 3. Materials and Methods

### 3.1. General Information

Solvents and reagents used for reactions were purchased from commercial suppliers. Catalyst Pd(PPh_3_)_4_ was purchased from Sigma-Aldrich (München, Germany). Solvents were dried under standard conditions; chemicals were used without further purification. 4-Bromo-2-iodoaniline [72], 1-(4-bromo-2-iodophenyl)-3-ethyl-3-phenyltriaz-1-ene **(1)** [40], Co_2_(CO)_6_-complex of diazacyclononyne **15** [69] and TBTA [67] were synthesized by known procedures without any modifications. Evaporation of solvents and concentration of reaction mixtures were performed in vacuum at 35 °C on a rotary evaporator. Thin-layer chromatography (TLC) was carried out on silica gel plates (Silica gel 60, F254, Merck (Darmstadt, Germany) with detection by UV or staining with a basic aqueous solution of KMnO_4_. Melting points (mp) determined are uncorrected. ^1^H and ^13^CNMR spectra were recorded at 400 and 100 MHz, respectively, at 25 °C in acetone-d_6_ without the internal standard using a 400 МHz Avance spectrometer (Bruker, Billerica, MA, USA). The ^1^H-NMR data are reported as chemical shifts (δ), multiplicity (s, singlet; d, doublet; t, triplet; q, quartet; m, multiplet; br, broad), coupling constants (J, given in Hz), and number of protons. The ^13^C NMR data are reported as the chemical shifts (δ) with coupling constant *J*(C−F) for F-containing compounds. Chemical shifts for ^1^H and ^13^C are reported as δ values (ppm) and referenced to residual solvent (δ = 2.05 ppm for ^1^H; δ = 29.84 for ^13^C–for spectra in acetone-*d_6_*, δ = 7.26 ppm for ^1^H; δ = 77.16 ppm for ^13^C–for spectra in CDCl_3_ and δ = 2.50 ppm for ^1^H; δ = 39.52 ppm for ^13^C–for spectra in DMSO-*d_6_*). High resolution mass spectra (HRMS) were determined using electrospray ionization (ESI) in the mode of positive ion registration with a Bruker microTOF mass analyzer (Billerica, MA, USA). UV–vis spectra for solutions of all compounds in THF were recorded on a UV-1800 spectrophotometer (Shimadzu, Kyoto, Japan) at 20 °C. Fluorescence spectra for the same solutions were recorded on a FluoroMax-4 spectrofluorometer (Horiba Scientific, Glasgow, Scotland) at 20 °C. IR spectra were measured using a Nicolet 8700 spectrometer (Thermo Scientific, Madison, WI, USA) equipped with a Thermo Scientific Smart iTR™ as an Attenuated Total Reflectance (ATR) sampling accessory. Data for **11f** were collected using an XtaLAB SuperNova diffractometer (Rigaku Oxford Diffraction, Tokio, Japan) equipped with an HyPix3000 CCD area detector operated with monochromated microfocused CuKα radiation (λ[CuKα] = 1.54184 Å). All the data were integrated and corrected for background, Lorentz, and polarization effects by means of the CrysAlisPro (Tokyo, Japan) [73] program complex. Absorption correction was applied using the empirical spherical model within the CrysAlisPro program complex using spherical harmonics, implemented in the SCALE3 ABSPACK scaling algorithm. The unit-cell parameters were refined by the least-squares techniques. The structures were solved by direct methods and refined using the SHELX [74] program incorporated in the OLEX2 [75] program package.

### 3.2. Synthetic Methods

#### 3.2.1. The Richter-Type Cyclization Protocol of Triazenes **3, 8**

To a solution of triazene **3** or **8** (1 equiv) in acetone (C = 0.1 M) HBr (48% aqueous solution, 20 equiv) was quickly added dropwise while maintaining the reaction mixture temperature of 20 °C by cooling the reaction mixture with a water bath. The resulting mixture was stirred for 10 minutes. Upon completion of the reaction, the reaction mixture was diluted with an aqueous solution of triethylamine (21 equiv). The resulting mixture was extracted with ethyl acetate, the combined organic layers were washed with water, brine and dried over anhydrous Na_2_SO_4_. The solvent was removed in vacuum and the crude 4-bromocinnoline was purified by column chromatography.

#### 3.2.2. General Procure for the Suzuki Coupling (Method A)

To a solution of triazene **3** or azidocinnoline **5** (1 equiv) in a mixture of toluene/1,4-dioxane/water (1:2:2) (C = 0.1 M) in a vial was added arylboronic acid **6** (1.5 equiv), Pd(PPh_3_)_4_ (5 mol%) and Na_2_CO_3_ (2 equiv). The vial was sealed; the reaction mixture was evacuated and flushed with Ar several times. The vial with the reaction mixture was placed in a preheated oil bath (80−100 °C) and stirred for 1−24 h (TLC control). After completion the reaction, the mixture was cooled and poured into a saturated aqueous solution of NH_4_Cl and extracted with ethyl acetate. The combined organic layers were washed with saturated aqueous solutions of NH_4_Cl and brine, dried over anhydrous Na_2_SO_4_, and concentrated under reduced pressure to yield the crude product, which was purified by column chromatography on silica gel.

#### 3.2.3. General Procure for the Suzuki Coupling (Method B)

Triazene **3** or azidocinnoline **5** (1 equiv), ArB(OH)_2_ **6** (1.5 equiv), K_3_PO_4_ (2 equiv), and Pd(PPh_3_)_4_ (5 mol %) were placed in a vial. The vial was sealed, and the mixture was evacuated and flushed with Ar several times. 1,4-Dioxane (C = 0.1 M) was added, and the vial with the reaction mixture was placed in a preheated oil bath (80−100 °С) and stirred for 1−20 h (TLC control). After cooling to rt, the reaction mixture was filtered through a pad silica gel and washed with ethyl acetate. Solvents were removed under reduced pressure, and the crude product was purified by column chromatography on silica gel.

#### 3.2.4. General Procure for the Nucleophilic Substitution

Sodium azide (2–5 equiv) was added to a solution of bromocinnoline (1 equiv) in absolute DMF (C = 0.1 M). The mixture was degassed and stirred under argon at 50 °C for 24 h (TLC control). Upon completion the reaction, the reaction mixture was poured into water, extracted with ethyl acetate; the combined organic layers were washed three times with water and two times with brine, dried over anhydrous Na_2_SO_4_. The solvent was removed in vacuum to yield the crude product, which was purified by column chromatography on silica gel.

#### 3.2.5. General Procure for CuAAC Synthesis of 4-triazolylcinnolines

To a stirred mixture of a terminal alkynes (1 equiv) and 4-azidocinnolines (1 equiv) in a mixture of water/THF (1:1) were added sodium ascorbate (10 mol%) and CuSO_4_ × 5H_2_O (5 mol%). The reaction mixture was vigorously stirred at 30 °C until the completion of the reaction (TLC control). The resulting mixture was diluted with saturated aqueous solution of NH_4_Cl (10 mL) and extracted with ethyl acetate (3 × 15 mL). The combined organic layers were dried over anhydrous Na_2_SO_4_ and concentrated under reduced pressure to yield the crude product, which was purified by column chromatography on silica gel.

#### 3.2.6. Detailed Synthetic Procedures

*1-[4-Bromo-2-(hept-1-yn-1-yl)phenyl]-3-ethyl-3-phenyl-triaz-1-ene* (**3**). To a solution of 1-(4-bromo-2-iodophenyl)-3-ethyl-3-phenyltriaz-1-ene (**1**) (1 equiv, 860 mg, 2 mmol) in a mixture of triethylamine (8 mL) and THF (4 mL) degassed by freeze-pump-thaw cycling were added Pd(PPh_3_)_4_ (5 mol.%, 116 mg, 0.1 mmol), CuI (15 mol.%, 57.0 mg, 0.3 mmol). The reaction mixture was degassed once again. Then hept-1-yne **2** (1.5 equiv, 289 mg, 0.39 mL, 3 mmol) was added via a syringe and the reaction mixture was stirred at 40 °C for 5 hours (TLC control). Upon completion of the reaction, the reaction mixture was poured into a saturated aqueous solution of NH_4_Cl, extracted with ethyl acetate (3 х 50 mL); the combined organic layers were washed with saturated aqueous solutions of NH_4_Cl, brine and dried over anhydrous Na_2_SO_4_. The solvent was removed under reduced pressure and the crude product was purified by column chromatography, using hexane/EtOAc/Et_3_N (90:1:0.01) as the eluent to give **3** (600 mg, 75%) as an orange oil. ^1^Н-NMR (acetone-*d_6_*) δ 7.58 – 7.60 (m, 3 H), 7.52 – 7.41 (m, 4 H), 7.17 (t, *J* = 7.4 Hz, 1H), 4.43 (q, *J* = 7.0 Hz, 2H), 2.48 (t, *J* = 7.0 Hz, 2H), 1.62 (p, *J* = 7.1 Hz, 2H), 1.51 − 1.44 (m, 2H), 1.41 − 1.32 (m, 5H), 0.91 (t, *J* = 7.3 Hz, 3H). ^13^С-NMR (acetone-*d_6_*) 151.6, 144.9, 135.9, 132.2, 130.2, 124.8, 123.0, 120.3, 119.4, 118.0, 97.3, 77.9, 41.2, 31.9, 29.2, 22.9, 20.1, 14.3, 11.2. HRMS (ESI): calcd. for C_21_H_25_BrN_3_ [M + H]^+^, 398.1232; found 398.1240.

*4,6-Dibromo-3-pentylcinnoline* (**4**). This compound was synthesized according to the general procedure for the Richter cyclization from triazene **3** (219 mg, 0.55 mmol). Purification of the crude product by column chromatography using hexane/EtOAc (40:1) as the eluent gave 150 mg (76%) of the title compound. ^1^H-NMR (acetone-*d_6_*) δ 8.42 (d, *J* = 9.0 Hz, 1H), 8.36 (d, *J* = 1.9 Hz, 1H), 8.08 (dd, *J* = 9.0, 2.0 Hz, 1H), 3.45 – 3.36 (m, 2H), 1.98 – 1.86 (m, 2H), 1.52 – 1.38 (m, 4H), 0.92 (t, *J* = 7.1 Hz, 3H). ^13^C-NMR (acetone-*d_6_*) δ 158.6, 149.0, 135.0, 132.9, 128.8, 128.4, 128.1, 125.5, 36.8, 32.3, 29.4, 23.1, 14.3. HRMS (ESI): calcd. for C_13_H_14_N_2_Br_2_Na [M + Na]^+^, 378.9416; found 378.9426.

*4-Azido-6-bromo-3-pentylcinnoline* (**5**). This compound was synthesized according to the general procedure for the nucleophilic substitution from bromocinnoline **4** (804 mg, 2.25 mmol) and sodium azide (730 mg, 11.25 mmol) in absolute DMF (22.3 mL). Purification of the crude product by column chromatography using hexane/EtOAc (10:1) as the eluent gave azidocinnoline **5** (603 mg, 84%). ^1^H- NMR (CDCl_3_) δ 8.36 (d, *J* = 9.1 Hz, 1H), 8.32 (d, *J* = 2.1 Hz, 1H), 7.87 (dd, *J* = 9.0, 2.0 Hz, 1H), 3.40–3.32 (m, 2H), 1.96–1.84 (m, 2H), 1.54–1.35 (m, 4H), 0.93 (t, *J* = 7.1 Hz, 3H). ^13^C-NMR (CDCl_3_) δ 151.4, 148.4, 134.2, 131.7, 131.4, 126.3, 123.9, 121.8, 32.5, 31.7, 29.7, 22.6, 14.1. HRMS (ESI): calcd. for C_13_H_14_N_5_BrNa [M + Na]^+^, 342.0325; found 342.0336.

*3-Ethyl-1-[3-(hept-1-yn-1-yl)-[1,1′-biphenyl]-4-yl]-3-phenyltriaz-1-ene* (**8a**). (A) This compound was synthesized according to the general procedure for the Suzuki coupling (Method A) from triazene **3** (230 mg, 0.57 mmol) and phenylboronic acid **6a** (105 mg, 0.86 mmol) at 100 °C; reaction time − 24. Purification of the crude product by column chromatography using hexane/EtOAc (70:1) as the eluent gave **8a** (166 mg, 73%) as an orange oil. ^1^Н-NMR (acetone-*d_6_*) δ 7.73−7.60 (m, 7H), 7.50–7.36 (m, 5 H), 7.5 –7.37 (m, 5H), 7.16 (t, *J* = 7.4 Hz, 1H), 4.46 (q, *J* = 7.0 Hz, 2H), 2.50 (t, *J* = 7.0 Hz, 2H), 1.6–1.61 (m, 2H), 1.53–1.46 (m, 2H), 1.42–1.33 (m, 5H), 0.92 (t, *J* = 7.3 Hz, 3H). ^13^С-NMR (acetone-*d_6_*) δ 150.6, 144.1, 139.8, 138.8, 131.0, 129.3, 128.9, 127.5, 126.8, 126.6, 123.6, 120.7, 118.1, 116.8, 94.8, 78.4, 40.0, 31.0, 28.5, 22.0, 19.2, 13.4, 10.4. HRMS (ESI): calcd. for C_27_H_30_N_3_ [M + H]^+^, 396.2434; found: 396.2433.

*3-Ethyl-1-[3-(hept-1-yn-1-yl)-4′-(trifluoromethyl)-[1,1′-biphenyl]-4-yl]-3-phenyltriaz-1-ene* (**8b**). (A) This compound was synthesized by the general procedure for the Suzuki coupling (Method A) from triazene **3** (100 mg, 0.25 mmol) and 4-trifluoromethylphenylboronic acid **6b** (72 mg, 0.38 mmol) at 100 °C; reaction time–24 h. Purification of the crude product by column chromatography using hexane/EtOAc (80:1) as the eluent gave **8b** (75.0 mg, 65%) as an orange oil.

(B) This compound was synthesized by the general procedure for the Suzuki coupling (method B) from triazene **3** (300 mg, 0.754 mmol) and 4-trifluoromethylphenylboronic acid **6b** (72 mg, 0.38 mmol) **6b** (190 mg, 1.50 mmol) at 100 °C; reaction time – 24 h. Purification of the crude product by column chromatography using hexane/EtOAc (80:1) as the eluent gave **8b** (205 mg, 55%) as an orange oil. ^1^Н-NMR (acetone-*d_6_*) δ 7.93 (d, *J* = 8.2 Hz, 2H), 7.84−7.80 (m, 3H), 7.75–7.65 (m, 2H), 7.64–7.58 (m, 2H), 7.49–7.40 (m, 2H), 7.22–7.13 (m, 1H), 4.47 (q, *J* = 7.0 Hz, 2H), 2.51 (t, *J* = 7.0 Hz, 2H), 1.71 – 1.59 (m, 2H), 1.56−1.44 (m, 2H), 1.43–1.31 (m, 5H), 0.92 (t, *J* = 7.3 Hz, 3H). ^13^С-NMR (acetone-*d_6_*) δ 152.3, 145.0, 144.6, 138.0, 132.3, 130.2, 129.7 (q, ^2^*J_C-F_* = 32.3 Hz), 128.2, 128.0, 126.7 (q, ^3^*J_C-F_* = 3.8 Hz), 125.5 (q, ^1^*J_C-F_* = 270.5 Hz), 124.7, 121.8, 119.3, 117.9, 96.1, 79.1, 41.1, 31.9, 29.4, 22.9, 20.1, 14.3, 11.3. HRMS (ESI): calcd. for C_28_H_29_F_3_N_3_ [M + H]^+^, 464.2313; found: 464.2308.

*3-Ethyl-1-[3-(hept-1-yn-1-yl)-4'-methoxy-[1,1′-biphenyl]-4-yl]-3-phenyltriaz-1-ene* (**8c**). This compound was synthesized by the general procedure for the Suzuki coupling (method A) from triazene **3** (100 mg, 0.25 mmol) and 4-methoxyphenylboronic acid **6c** (58 mg, 0.38 mmol) at 100 °C; reaction time – 24 h. Purification of the crude product by column chromatography using hexane/EtOAc (80:1) as the eluent gave **8c** (66 mg, 62%) as a brown oil. ^1^Н-NMR (acetone-*d_6_*) δ 7.68 (d, *J* = 2.0 Hz, 1H), 7.66−7.54 (m, 6H), 7.48–7.38 (m, 2H), 7.17–7.13 (m, 1H), 7.07–6.99 (m, 2H), 4.45 (q, *J* = 7.0 Hz, 2H), 3.85 (s, 3H), 2.50 (t, *J* = 7.0 Hz, 2H), 1.70–1.58 (m, 2H), 1.55−1.43 (m, 2H), 1.43 – 1.30 (m, 5H), 0.91 (t, *J* = 7.3 Hz, 3H). ^13^С-NMR (acetone-*d_6_*) δ 160.5, 150.9, 145.1, 139.5, 133.0, 131.4, 130.2, 128.6, 127.3, 124.4, 121.6, 119.0, 117.7, 115.2, 95.5, 79.4, 55.7, 40.8, 31.9, 29.4, 22.9, 20.2, 14.3, 11.3. HRMS (ESI): calcd. for C_28_H_32_N_3_O [M + H]^+^, 426.2540; found 426.2525.

*3-Ethyl-1-[3-(hept-1-yn-1-yl)-4′-phenoxy-[1,1′-biphenyl]-4-yl]-3-phenyltriaz-1-ene* (**8d**). (A) This compound was synthesized by the general procedure for the Suzuki coupling (method A) from triazene **3** (100 mg, 0.25 mmol) and 4-phenoxyphenylboronic acid **6d** (81 mg, 0.38 mmol) at 100 °C; reaction time – 24 h. Purification of the crude product by column chromatography using hexane/EtOAc (70:1) as the eluent gave **8d** (68 mg, 56%) as a brown oil.

(B) This compound was synthesized by the general procedure for the Suzuki coupling (method B) from triazene **3** (300 mg, 0.75 mmol) and 4-phenoxyphenylboronic acid **6d** (323 mg, 1.51 mmol) at 100 °C; reaction time – 4 h. Purification of the crude product by column chromatography using hexane/EtOAc (80:1) as the eluent gave **8d** (265 mg, 72%) as a brown oil. ^1^Н-NMR (acetone-*d_6_*) δ 7.76–7.68 (m, 3H), 7.67−7.58 (m, 4H), 7.48–7.38 (m, 4H), 7.22–7.14 (m, 2H), 7.12–7.04 (m, 4H), 4.46 (q, *J* = 7.0 Hz, 2H), 2.50 (t, *J* = 7.0 Hz, 2H), 1.70–1.59 (m, 2H), 1.56−1.44 (m, 2H), 1.42–1.33 (m, 5H), 0.91 (t, *J* = 7.3 Hz, 3H). ^13^С-NMR (acetone-*d_6_*) δ 158.1, 158.0, 151.3, 145.1, 139.1, 135.8, 131.7, 130.9, 130.2, 129.1, 127.5, 124.5, 124.5, 121.7, 119.9, 119.9, 119.1, 117.7, 95.7, 79.3, 40.9, 31.9, 23.0, 20.2, 14.3, 11.3. HRMS (ESI): calcd. for C_33_H_33_N_3_ONa [M + Na]^+^, 510.2516; found 510.2521.

*tret-Butyl [4′-(3-ethyl-3-phenyltriaz-1-en-1-yl)-3′-(hept-1-yn-1-yl)-[1,1′-biphenyl]-4-yl]carbamate* (**8e**). This compound was synthesized by the general procedure for the Suzuki coupling (method B) from triazene **3** (360 mg, 0.905 mmol) and {4-[(*tert*-butoxycarbonyl)amino]phenyl}boronic acid **6e** (430 mg, 1.8 mmol) at 100 °C; reaction time – 2.5 h. Purification of the crude product by column chromatography using hexane/EtOAc (15:1) as the eluent gave **8e** (345 mg, 75%) as an yellow oil. ^1^Н-NMR (acetone-*d_6_*) δ 8.49 (s, 1H), 7.72–7.56 (m, 9H), 7.48–7.39 (m, 2H), 7.20–7.10 (m, 1H), 4.45 (q, *J* = 7.0 Hz, 2H), 2.50 (t, *J* = 7.0 Hz, 2H), 1.69–1.59 (m, 2H), 1.54−1.45 (m, 11H), 1.42–1.32 (m, 5H), 0.91 (t, *J* = 7.3 Hz, 3H). ^13^С-NMR (acetone-*d_6_*) δ 153.7, 151.0, 145.1, 140.3, 139.4, 134.5, 131.4, 130.2, 127.8, 127.2, 124.4, 121.6, 119.5, 119.0, 117.7, 95.5, 80.1, 79.4, 40.8, 31.9, 29.4, 28.6, 22.9, 20.1, 14.3, 11.3. HRMS (ESI): calcd. for C_32_H_38_N_4_O_2_ [M + H]^+^, 511.3068; found 511.3086.

*4′-(3-Ethyl-3-phenyltriaz-1-en-1-yl)-3′-(hept-1-yn-1-yl)-[1,1′-biphenyl]-4-carbonitrile* (**8f**). This compound was synthesized by the general procedure for the Suzuki coupling (method B) from triazene **3** (340 mg, 0.854 mmol) and 4-cyanophenylboronic acid **6f** (251 mg, 1.7 mmol) at 80 °C; reaction time – 10 h. Purification of the crude product by column chromatography using hexane/EtOAc (30:1) as the eluent gave **8f** (190 mg, 53%) as an yellow oil. ^1^H-NMR (acetone-*d_6_*) δ 7.96–7.83 (m, 4H), 7.79 (d, *J* = 2.0 Hz, 1H), 7.76–7.63 (m, 2H), 7.62–7.56 (m, 2H), 7.50–7.38 (m, 2H), 7.21–7.15 (m, 1H), 4.45 (q, *J* = 7.1 Hz, 2H), 2.49 (t, *J* = 7.0 Hz, 2H), 1.66–1.59 (m, 2H), 1.55–1.41 (m, 2H), 1.40–1.31 (m, 5H), 0.89 (t, *J* = 7.3 Hz, 3H). ^13^C-NMR (acetone-*d_6_*) δ 152.5, 145.1, 145.0, 137.6, 133.6, 132.4, 130.2, 128.4, 128.0, 124.8, 121.9, 119.4, 119.3, 117.9, 111.8, 96.2, 79.0, 41.1, 31.9, 22.9, 20.1, 14.3, 11.3. HRMS (ESI): calcd. for C_28_H_28_N_4_Na [M + Na]^+^, 443.2206; found 443.2191.

*3-Ethyl-1-(2-(hept-1-yn-1-yl)-4-(thiophen-3-yl)phenyl)-3-phenyltriaz-1-ene* (**8g**). This compound was synthesized by the general procedure for the Suzuki coupling (method B) from triazene **3** (330 mg, 0.83 mmol) and thiophen-3-ylboronic acid **6g** (286 mg, 1.66 mmol) at 100 °C; reaction time – 4 h. Purification of the crude product by column chromatography using hexane/EtOAc (30:1) as the eluent gave **8g** (316 mg, 95%) as a yellow oil. ^1^H-NMR (acetone-*d_6_*) δ 7.79–7.78 (m, 2H), 7.68 (dd, *J* = 8.5, 2.1 Hz, 1H), 7.64–7.51 (m, 5H), 7.46–7.37 (m, 2H), 7.17–7.13 (m, 1H), 4.44 (q, *J* = 7.0 Hz, 2H), 2.49 (t, *J* = 7.0 Hz, 2H), 1.70–1.58 (m, 2H), 1.56–1.43 (m, 2H), 1.41–1.32 (m, 5H), 0.91 (t, *J* = 7.3 Hz, 3H). ^13^C-NMR (acetone-*d_6_*) δ 151.2, 145.0, 141.9, 134.7, 131.2, 130.2, 127.5, 127.1, 126.9, 124.5, 121.7, 121.5, 119.0, 117.7, 95.7, 79.3, 40.9, 31.9, 29.4, 22.9, 20.2, 14.3, 11.3. HRMS (ESI): calcd. for C_25_H_27_N_3_SCu [M + Cu]^+^, 464.1216; found 464.1194.

*4-Bromo-3-pentyl-6-phenylcinoline* (**9a**). This compound was synthesized by the general method for the Richter cyclization from triazene **8a** (200 mg, 0.505 mmol). Purification of the crude product by column chromatography using hexane/EtOAc (10:1→7:1) as the eluent gave **9a** (153 mg, 86%) as a yellow oil. ^1^H-NMR (acetone-*d_6_*) δ 8.51 (d, *J* = 8.8 Hz, 1H), 8.30–8.21 (m, 2H), 7.89–7.84 (m, 2H), 7.60–7.47 (m, 3H), 3.40–3.35 (m, 2H), 1.95–1.87 (m, 2H), 1.51–1.36 (m, 4H), 0.92 (t, *J* = 7.1 Hz, 3H). ^13^C-NMR (acetone-*d_6_*) δ 158.0, 149.8, 145.6, 139.8, 131.4, 131.1, 130.2, 129.8, 128.6, 127.5, 127.1, 123.5, 36.8, 32.3, 29.4, 23.1, 14.3. HRMS (ESI): calcd. for C_19_H_20_N_2_Br [M + H]^+^, 355.0804; found 355.0802.

*4-Bromo-3-pentyl-6-[4-(trifluoromethyl)phenyl]cinnoline* (**9b**). This compound was synthesized by the general method for the Richter cyclization from triazene **8b** (50 mg, 0.108 mmol). Purification of the crude product by column chromatography using hexane/EtOAc (20:1) as the eluent gave **9b** (40 mg, 88%), m.p. 88–89 °C. ^1^H-NMR (acetone-*d_6_*) δ 8.58 (d, *J* = 8.8 Hz, 1H), 8.39 (d, *J* = 1.6 Hz, 1H), 8.30 (dd, *J* = 8.8, 1.9 Hz, 1H), 8.12 (d, *J* = 8.1 Hz, 2H), 7.92 (d, *J* = 8.2 Hz, 2H), 3.42–3.39 (m, 2H), 2.00–1.86 (m, 2H), 1.53–1.36 (m, 4H), 0.93 (t, *J* = 7.1 Hz, 3H). ^13^C-NMR (100 MHz, acetone-*d_6_*) δ 158.3, 149.9, 144.1, 143.8, 131.7, 131.2 (q, ^2^*J_C-F_* = 32.3 Hz), 131.0, 129.5, 127.4, 127.3, 127.0 (q, ^3^*J_C-F_* = 3.4 Hz), 126.7 (q, ^1^*J_C-F_* = 271.5 Hz), 124.6, 36.9, 32.3, 29.4, 23.1, 14.3. HRMS (ESI): calcd. for C_20_H_18_BrF_3_N_2_Na [M + Na]^+^, 445.0498; found 445.0480.

*4-Bromo-6-(4-methoxyphenyl)-3-pentylcinnoline* (**9c**). This compound was synthesized by the general method for the Richter cyclization from triazene **8c** (228 mg, 0.536 mmol). Purification of the crude product by column chromatography using hexane/EtOAc (10:1→5:1) as the eluent gave **9c** (157 mg, 76%), m.p. 85–87 °C. ^1^H-NMR (acetone-*d_6_*) δ 8.52–8.45 (m, 1H), 8.25–8.21 (m, 2H), 7.88–7.82 (m, 2H), 7.17–7.09 (m, 2H), 3.90 (s, 3H), 3.43–3.30 (m, 2H), 1.99–1.86 (m, 2H), 1.54–1.36 (m, 4H), 0.93 (t, *J* = 7.1 Hz, 3H). ^13^C-NMR (acetone-*d_6_*) δ 161.7, 157.9, 149.7, 145.3, 132.0, 131.3, 130.9, 129.9, 127.7, 126.9, 122.3, 115.7, 55.8, 36.9, 32.3, 23.2, 14.3. HRMS (ESI): calcd. for C_20_H_21_BrN_2_ONa [M + Na]^+^, 407.0729; found 407.0708.

*4-Bromo-3-pentyl-6-(4-phenoxyphenyl)cinnoline* (**9d**). This compound was synthesized by the general method for the Richter cyclization from triazene **8d** (180 mg, 0.37 mmol). Purification of the crude product by column chromatography using hexane/EtOAc (10:1→5:1) as the eluent gave **9d** (121 mg, 72%), m.p. 77–78 °C. ^1^H-NMR (400 MHz, acetone-*d_6_*) δ = 8.51 (dd, *J* = 8.8, 0.7 Hz, 1H), 8.31–8.21 (m, 2H), 7.95–7.87 (m, 2H), 7.50–7.40 (m, 2H), 7.26–7.08 (m, 5H), 3.43–3.34 (m, 2H), 1.99–1.86 (m, 2H), 1.54–1.35 (m, 4H), 0.92 (t, *J* = 7.1 Hz, 3H). ^13^C-NMR (100 MHz, acetone-*d_6_*) δ 159.5, 158.0, 157.5, 149.7, 144.9, 134.6, 131.4, 131.0, 130.9, 130.3, 127.6, 127.0, 124.9, 122.9, 120.3, 119.8, 36.8, 32.3, 23.1, 14.3. HRMS (ESI): calcd. for C_25_H_23_BrN_2_ONa [M + Na]^+^, 469.0886; found 469.0897.

*tert-Butyl [4-(4-bromo-3-pentylcinnolin-6-yl)phenyl]carbamate* (**9e**). This compound was synthesized by the general method for the Richter cyclization from triazene **8e** (170 mg, 0.333 mmol). Purification of the crude product by column chromatography using hexane/EtOAc (5:1) as the eluent gave **9e** (135 mg, 86%). ^1^H-NMR (acetone-*d_6_*) δ 8.66 (s, 1H), 8.53–8.45 (m, 1H), 8.31–8.22 (m, 2H), 7.89–7.74 (m, 4H), 3.43–3.34 (m, 2H), 1.99–1.86 (m, 2H), 1.52 (s, 9H), 1.51–1.37 (m, 5H), 0.93 (t, *J* = 7.0 Hz, 3H). ^13^C NMR (acetone-*d_6_*) δ 158.0, 153.7, 149.8, 145.3, 141.8, 133.3, 131.3, 130.8, 129.0, 127.7, 126.9, 122.3, 119.6, 80.4, 36.8, 32.3, 28.5, 23.1, 14.3. HRMS (ESI): calcd. for C_24_H_28_BrN_3_O_2_Na [M + Na]^+^, 492.1257; found 492.1262. 

*4-(4-Bromo-3-pentylcinnolin-6-yl)benzonitrile* (**9f**). This compound was synthesized by the general method for Richter cyclization from triazene **8f** (190 mg, 0.452 mmol). Purification of the crude product by recrystallization from hexane/EtOAc (1:1) gave **9f** (107 mg, 62%) as a brown solid. ^1^H-NMR (acetone-*d_6_*) δ 8.60 (d, *J* = 8.8 Hz, 1H), 8.44–8.40 (m, 1H), 8.32 (dd, *J* = 8.8, 1.9 Hz, 1H), 8.15–8.10 (m, 2 H), 8.02–7.97 (m, 2H), 3.45–3.38 (m, 2H), 1.98–1.87 (m, 2H), 1.54–1.34 (m, 4H), 0.93 (t, *J* = 7.0 Hz, 3H).^13^C-NMR (acetone-*d_6_*) δ 152.4, 149.9, 144.2, 143.8, 133.9, 133.7, 131.8, 130.9, 129.6, 127.3, 124.9, 119.3, 113.4, 36.8, 32.3, 29.4, 23.1, 14.3. HRMS (ESI): calcd. for C_20_H_18_BrN_3_Na [M + Na]^+^, 402.0576; found 402.0583.

*4-Bromo-3-pentyl-6-(thiophen-3-yl)cinnoline* (**9g**). This compound was synthesized by the general method for the Richter cyclization from triazene **8g** (280 mg, 0.698 mmol). Purification of the crude product by column chromatography using hexane/EtOAc (10:1) as the eluent gave **9g** (214 mg, 85%). ^1^H-NMR (acetone-*d_6_*) δ 8.47–8.40 (m, 1H), 8.33–8.25 (m, 2H), 8.14 (dd, *J* = 2.9, 1.4 Hz, 1H), 7.74 (dd, *J* = 5.1, 1.4 Hz, 1H), 7.68 (dd, *J* = 5.1, 2.9 Hz, 1H), 3.39–3.31 (m, 2H), 1.95–1.85 (m, 2H), 1.50–1.36 (m, 4H), 0.91 (t, *J* = 7.1 Hz, 3H). ^13^C-NMR (acetone-*d_6_*) δ 158.0, 149.7, 141.0, 140.1, 131.3, 130.6, 128.5, 127.8, 127.1, 126.9, 125.2, 122.0, 36.8, 32.3, 29.4, 23.1, 14.3. HRMS (ESI): calcd. for C_17_H_17_BrN_2_SNa [M + Na]^+^, 383.0188; found 383.0188.

*4-Azido-3-pentyl-6-phenylcinoline* (**7a**). (A) This compound was synthesized by the general procedure for the Suzuki coupling (method A) from 4-azido-6-bromocinnoline **5** (50 mg, 0.156 mmol) and phenylboronic acid **6a** (38 mg, 0.312 mmol) at 80 °C; reaction time – 1 h. Purification of the crude product by column chromatography using hexane/EtOAc (5:1→2:1→1:2) as the eluent gave **7a** (9,5 mg, 19%). (B) Azidocinnoline **7a** was obtained by the general procedure for the Suzuki coupling (method B) from 4-azido-6-bromcinnoline **5** (50 mg, 0.156 mmol), phenylboronic acid **6a** (38 mg, 0.312 mmol) and K_3_PO_4_ (99 mg, 0.468 mmol, 3 equiv) at 80 °C; reaction time–1 h. Purification of the crude product by column chromatography using hexane/EtOAc (5:1→2:1→1:2) as the eluent gave **7a** (25 mg, 50%). С) Azidocinnoline **7a** was prepared by the general procure for the nucleophilic substitution from bromocinnoline **9a** (650 mg, 1.83 mmol) and sodium azide (594 mg, 9.15 mmol). Purification of the crude product by column chromatography using hexane/EtOAc (8:1) as the eluent gave **7a** (528 mg, 91%). ^1^H-NMR (acetone-*d_6_*) δ 8.49 (d, *J* = 8.9 Hz, 1H), 8.41 (d, *J* = 1.6 Hz, 1H), 8.23 (dd, *J* = 8.9, 1.6 Hz, 1H), 7.91–7.84 (m, 2H), 7.63–7.53 (m, 2H), 7.52–7.48 (m, 1H), 3.41–3.28 (m, 2H), 1.97–1.90 (m, 2H), 1.57–1.35 (m, 4H), 0.93 (t, *J* = 7.1 Hz, 3H). ^13^C-NMR (acetone-*d_6_*) δ 151.6, 150.3, 144.1, 140.2, 133.2, 131.2, 130.8, 130.2, 129.7, 128.5, 121.8, 119.3, 32.9, 32.4, 23.2, 14.3. HRMS (ESI): calcd. for C_19_H_20_N_5_ [M + H]^+^, 318.1713; found 318.1714. IR, cm^−1^, *ν*: 2117 (N_3_).

*4-Azido-3-pentyl-6-(4-(trifluoromethyl)phenyl)cinnoline* (**7b**). This compound was synthesized by the general procure for the nucleophilic substitution from bromocinnoline **9b** (130 mg, 0.308 mmol) and sodium azide (100 mg, 1.54 mmol). Purification of the crude product by column chromatography using hexane/EtOAc (3:1) as the eluent gave **7b** (100 mg, 84%). ^1^H-NMR (acetone-*d_6_*) δ 8.57–8.48 (m, 2H), 8.27 (dd, *J* = 8.8 Hz, 2.0 Hz, 1H), 8.11 (d, *J* = 8.1 Hz, 2H), 7.92 (d, *J* = 8.1 Hz, 2H), 3.42–3.33 (m, 2H), 2.00–1.88 (m, 2H), 1.54–1.38 (m, 4H), 0.93 (t, *J* = 7.0 Hz, 3H). ^13^C-NMR (acetone-*d_6_*) δ 151.8, 150.3, 144.1, 142.5, 133.4, 131.5, 130.9 (q, ^2^*J_C–F_* = 32.4 Hz), 130.6, 129.3, 127.0 (q, ^3^*J_C–F_* = 3.8 Hz), 125.4 (q, ^1^*J_C–F_* = 271.1 Hz), 121.6, 120.5, 32.9, 32.4, 30.0, 23.2, 14.3 ppm. HRMS (ESI): calcd. for C_20_H_18_F_3_N_5_Na [M + Na]^+^, 408.1407; found 408.1403. IR, cm^−1^, *ν*: 2122 (N_3_).

*4-Azido-6-(4-methoxyphenyl) -3-pentylcinnoline* (**7c**). This compound was synthesized by the general procure for the nucleophilic substitution from bromocinnoline **9c** (155 mg, 0.404 mmol) and sodium azide (65.7 mg, 1.01 mmol). Purification of the crude product by column chromatography using hexane/EtOAc (5:1→3:1) as the eluent gave **7c** (116 mg, 83%). ^1^H-NMR (acetone-*d_6_*) δ 8.44 (d, *J* = 8.9 Hz, 1H), 8.33 (d, *J* = 2.0 Hz, 1H), 8.20 (dd, *J* = 8.9, 1.9 Hz, 1H), 7.88–7.77 (m, 2H), 7.22–7.08 (m, 2H), 3.89 (s, 3H), 3.38–3.21 (m, 2H), 1.96–1.88 (m, 2H), 1.53–1.40 (m, 4H), 0.93 (t, *J* = 7.1 Hz, 3H). ^13^C-NMR (acetone-*d_6_*) δ 161.6, 151.5, 150.2, 143.8, 132.9, 132.2, 131.1, 130.5, 129.7, 121.9, 118.0, 115.6, 55.8, 32.9, 32.4, 30.0, 23.2, 14.3. HRMS (ESI): calcd. for C_20_H_21_N_5_ONa [M + Na]^+^, 370.1638; found 370.1626. IR, cm^−1^, *ν*: 2116 (N_3_).

*4-Azido-3-pentyl-6-(4-phenoxyphenyl)cinnoline* (**7d**). This compound was synthesized by the general procure for the nucleophilic substitution from bromocinnoline **9d** (120 mg, 0.269 mmol) and sodium azide (87 mg, 1.35 mmol). Purification of the crude product by column chromatography using hexane/EtOAc (5:1→ 3:1) as the eluent gave **7d** (77 mg, 70%). ^1^H-NMR (acetone-*d_6_*) δ 8.47 (d, *J* = 8.9 Hz, 1H), 8.38 (d, *J* = 1.8 Hz, 1H), 8.22 (dd, *J* = 8.9, 1.9 Hz, 1H), 7.94–7.86 (m, 2H), 7.49 – 7.40 (m, 2H), 7.25–7.13 (m, 3H), 7.16–7.08 (m, 2H), 3.39–3.30 (m, 2H), 1.99–1.87 (m, 2H), 1.53–1.39 (m, 4H), 0.93 (t, *J* = 7.0 Hz, 3H). ^13^C-NMR (acetone-*d_6_*) δ 159.4, 157.6, 151.6, 150.2, 143.4, 134.9, 133.1, 131.2, 131.0, 130.9, 130.6, 130.1, 124.9, 121.8, 120.3, 119.8, 119.7, 118.7, 32.9, 32.4, 30.0, 23.2, 14.3. HRMS (ESI): calcd. for C_25_H_23_N_5_ONa [M + Na]^+^, 432.1795; found 432.1797.

*tert-Butyl (4-(4-azido-3-pentylcinnolin-6-yl)phenyl)carbamate* (**7e**). This compound was synthesized by the general procure for the nucleophilic substitution from bromocinnoline **9e** (88 mg, 0.188 mmol) and sodium azide (61 mg, 0.94 mmol). Purification of the crude product by column chromatography using hexane/EtOAc (2:1→ 1:1) as the eluent gave **7e** (59 mg, 73%). ^1^H-NMR (acetone-*d_6_*) δ 8.65 (s, 1H), 8.46 (d, *J* = 8.9 Hz, 1H), 8.38 (d, *J* = 1.7 Hz, 1H), 8.23 (dd, *J* = 8.9, 1.8 Hz, 1H), 7.86–7.73 (m, 4H), 3.39–3.29 (m, 2H), 1.98–1.87 (m, 2H), 1.52 (s, 9H), 1.49–1.40 (m, 4H), 0.93 (t, *J* = 7.0 Hz, 3H). ^13^C-NMR (acetone-*d_6_*) δ = 153.7, 153.6, 151.5, 150.2, 143.7, 141.7, 133.6, 133.0, 131.1, 130.5, 128.9, 121.9, 119.6, 119.5, 118.1, 80.4, 32.9, 32.4, 29.8, 28.5, 23.2, 14.3. HRMS (ESI): calcd. for C_24_H_29_N_6_O_2_ [M + H]^+^, 433.2347; found 433.2342.

*4-(4-Azido-3-pentylcinnolin-6-yl)benzonitrile* (**7f**). This compound was synthesized by the general procure for the nucleophilic substitution from bromocinnoline **9f** (100 mg, 0.264 mmol) and sodium azide (86 mg, 1.32 mmol). Purification of the crude product by column chromatography using hexane/EtOAc (5:1) as the eluent gave **7f** (80 mg, 88%). ^1^H-NMR (acetone-*d_6_*) δ 8.57–8.47 (m, 2H), 8.27 (dd, *J* = 8.9, 2.0 Hz, 1H), 8.15–8.04 (m, 2H), 8.02–7.92 (m, 2H), 3.46–3.22 (m, 2H), 2.00–1.88 (m, 2H), 1.56–1.36 (m, 4H), 0.93 (t, *J* = 7.1 Hz, 3H). ^13^C-NMR (acetone-*d_6_*) δ 151.9, 150.3, 144.5, 142.1, 133.9, 133.5, 131.5, 130.4, 129.5, 121.6, 120.7, 119.1, 113.2, 32.9, 32.4, 29.1, 23.2, 14.3. HRMS (ESI): calcd. for C_20_H_18_N_6_Na [M + Na]^+^, 365.1485; found 365.1472. 

*4-Azido-3-pentyl-6-(thiophen-3-yl)cinnoline* (**7g**). This compound was synthesized by the general procure for the nucleophilic substitution from bromocinnoline **9g** (185 mg, 0.514 mmol) and sodium azide (167 mg, 2.57 mmol). Purification of the crude product by column chromatography using hexane/EtOAc (7:1→3:1) as the eluent gave **7g** (115 mg, 69%), m.p. 100–102 °C. ^1^H-NMR (acetone-*d_6_*) δ 8.46–8.39 (m, 2H), 8.29 (dd, *J* = 8.9, 1.9 Hz, 1H), 8.13 (dd, *J* = 2.9, 1.4 Hz, 1H), 7.76 (dd, *J* = 5.1, 1.4 Hz, 1H), 7.70 (dd, *J* = 5.1, 2.9 Hz, 1H), 3.37–3.29 (m, 2H), 1.98–1.86 (m, 2H), 1.54–1.37 (m, 4H), 0.93 (t, *J* = 7.1 Hz, 3H). ^13^C-NMR (acetone-*d_6_*) δ 151.6, 150.2, 141.3, 138.7, 133.0, 131.2, 130.3, 128.4, 127.1, 124.7, 122.0, 117.9, 32.9, 32.4, 30.0, 23.2, 14.3. HRMS (ESI): calcd. for C_17_H_17_N_5_SNa [M + Na]^+^, 346.1097; found 346.1085.

*3-[1-(3-Pentyl-6-phenylcinnolin-4-yl)-1H-1,2,3-triazol-4-yl]propan-1-ol* (**11a**). This compound was synthesized in accordance with the general procedure from pent-4-yn-1-ol **10a** (47.8 mg, 0.568 mmol), 4-azidocinnoline **7a** (90.0 mg, 0.284 mmol), CuSO_4_∙5H_2_O (3.5 mg, 0.014 mmol), and sodium ascorbate (5.60 mg, 0.028 mmol) in a mixture of THF/H_2_O (5.6 mL); reaction time – 5 h. The crude product was purified by column chromatography (eluent: hexane/EtOAc = 2:1) to afford triazole **11a** as a yellowish solid (100 mg, 88%), m.p. 89−91 °C. ^1^H-NMR (acetone*-d_6_)* δ 8.67 (d, *J* = 8.9 Hz, 1H), 8.35 (s, 1H), 8.30 (dd, *J* = 8.9, 1.9 Hz, 1H), 7.75–7.68 (m, 2H), 7.56–7.42 (m, 4H), 3.74–3.67 (m, 3H), 3.04–2.95 (m, 4H), 2.03–1.98 (m, 2H), 1.83–1.71 (m, 2H), 1.34–1.24 (m, 4H), 0.88–0.80 (m, 3H). ^13^C-NMR (acetone-*d_6_*) δ 154.3, 150.8, 149.2, 146.0, 139.7, 131.3, 131.2, 130.4, 130.1, 129.9, 128.5, 125.8, 123.6, 119.1, 61.6, 33.3, 32.5, 32.2, 30.0, 22.9, 22.7, 14.2. HRMS ESI: [M + H]^+^ calcd. for C_24_H_27_N_5_O^+^: 402.2288; found: 402.2293.

*4-[4-(3,4-Dimethoxyphenyl)-1H-1,2,3-triazol-1-yl]-3-pentyl-6-[4-(trifluoromethyl)phenyl]cinnoline* (**11b**). This compound was synthesized in accordance with the general procedure from 4-ethynyl-1,2-dimethoxybenzene **10b** (21.0 mg, 0.130 mmol), 4-azidocinnoline **7b** (50.0 mg, 0.130 mmol), CuSO_4_∙5H_2_O (1.7 mg, 0.007 mmol), and sodium ascorbate (2.6 mg, 0.013 mmol) in a mixture of THF/H_2_O (5 mL); reaction time – 3 h. The crude product was purified by column chromatography (eluent: hexane/EtOAc = 2:1) to afford triazole **11b** as a yellowish solid (36 mg, 50%). ^1^H-NMR (acetone-*d_6_*) δ 8.92 (s, 1H), 8.76 (d, *J* = 8.9 Hz, 1H), 8.37 (dd, *J* = 8.9, 1.7 Hz, 1H), 7.98 (d, *J* = 8.5 Hz, 2H), 7.86–7.79 (m, 3H), 7.66 (d, *J* = 2.0 Hz, 1H), 7.58 (dd, *J* = 8.3, 2.0 Hz, 1H), 7.08 (d, *J* = 8.3 Hz, 1H), 3.90 (s, 3H), 3.87 (s, 3H), 3.11–3.03 (m, 2H), 1.83 (p, *J* = 7.2 Hz, 2H), 1.36–1.23 (m, 4H), 0.83 (t, *J* = 7.0 Hz, 3H). ^13^C-NMR (acetone-*d_6_*) δ 154.5, 150.88, 150.87, 150.82, 149.0, 144.6, 143.6 (q, ^5^*J_C–F_* = 1.2 Hz), 131.6, 131.32, 131.1 (q, ^2^*J_C–F_* = 32.4 Hz), 130.3, 129.5, 126.9 (q, ^3^*J_C–F_* = 3.8 Hz), 125.2 (q, ^1^*J_C–F_* = 271.4 Hz), 124.06, 124.05, 123.3, 120.4, 119.4, 113.2, 110.6, 56.25, 56.24, 32.6, 32.2, 30.0, 22.9, 14.2. HRMS (ESI): calcd. for C_30_H_28_N_5_O_2_F_3_Na [M + Na]^+^; 570.2087; found: 570.2081.

*6-(4-Methoxyphenyl)-4-[4-(4-methoxyphenyl)-1H-1,2,3-triazol-1-yl]-3-pentylcinnoline* (**11c**). (A) This compound was prepared in accordance with the general procedure (method A) from 4-ethynyl-1-methoxybenzene **10c** (13.8 mg, 0.104 mmol), 4-azidocinnoline **7c** (40.0 mg, 0.104 mmol), CuSO_4_∙5H_2_O (1.3 mg, 0.005 mmol), and sodium ascorbate (2.10 mg, 0.010 mmol) in a mixture of THF/H_2_O (4 mL); reaction time − 2 h. The crude product was purified by column chromatography (eluent: hexane/EtOAc = 3:1) to afford triazole **11c** as a yellowish solid (35 mg, 76%). (B) This compound was prepared in accordance with the general procedure (method A) from mixture 4-ethynyl-1-methoxybenzene **10c** (24.0 mg, 0.177 mmol), 4-azidocinnoline **7c** (68.0 mg, 0.177 mmol), CuSO_4_∙5H_2_O (2.5 mg, 0.009 mmol), and sodium ascorbate (3.6 mg, 0.009 mmol) in the mixture of THF/H_2_O (4 mL) with the addition of TBTA (5.0 mg, 0.009 mmol); reaction time − 50 h. The crude product was purified by column chromatography (eluent: hexane/EtOAc = 3:1) to afford triazole **11c** as a solid (30 mg, 35%).^1^H-NMR (CDCl_3_) δ 8.68 (d, *J* = 8.9 Hz, 1H), 8.11 (dd, *J* = 8.9, 1.9 Hz, 1H), 8.01 (s, 1H), 7.94–7.88 (m, 2H), 7.58–7.52 (m, 2H), 7.44 (d, *J* = 1.8 Hz, 1H), 7.07–7.03 (m, 2H), 7.00–6.95 (m, 2H), 3.89 (s, 3H), 3.84 (s, 3H), 3.07–2.98 (m, 2H), 1.90–1.82 (m, 2H), 1.35–1.28 (m, 4H), 0.85 (t, *J* = 6.8 Hz, 4H). ^13^C-NMR (CDCl_3_) δ 160.8, 160.3, 153.7, 150.0, 148.5, 145.6, 131.2, 130.7, 130.5, 129.1, 127.5, 123.2, 122.4, 121.5, 117.0, 114.9, 114.7, 55.6, 32.0, 31.7, 29.8, 22.4, 14.0. HRMS (ESI): calcd. for C_29_H_29_N_5_O_2_Na [M + Na]^+^; 502.2213; found: 502.2227.

*4{1-[3-Pentyl-6-(4-phenoxyphenyl)cinnolin-4-yl]-1H-1,2,3-triazol-4-yl}butan-1-ol* (**11d**). This compound was prepared in accordance with the general procedure from hex-5-yn-1-ol **10d** (3.60 mg, 0.037 mmol), 4-azido-3-pentyl-6-(4-phenoxyphenyl)cinnoline **7d** (15.0 mg, 0.037 mmol), CuSO_4_∙5H_2_O (0.46 mg, 0.0018 mmol), and sodium ascorbate (0.67 mg, 0.0037 mmol) in a mixture of THF/H_2_O (1.5 mL); reaction time − 21 h. The crude product was purified by column chromatography (eluent: hexane/acetone = 2:1) to afford triazole **11d** as a brown oil (5 mg, 28%). ^1^H-NMR (DMSO-*d_6_*) δ 8.71 (d, *J* = 8.9 Hz, 1H), 8.55 (s, 1H), 8.35 (dd, *J* = 8.9, 1.9 Hz, 1H), 7.78–7.72 (m, 2H), 7.48–7.40 (m, 2H), 7.38 (d, *J* = 1.9 Hz, 1H), 7.23–7.17 (m, 1H), 7.15–7.04 (m, 4H), 4.41 (t, *J* = 5.1 Hz, 1H, OH), 3.45 (td, *J* = 6.4, 5.1 Hz, 2H), 2.94–2.90 (m, 2H), 2.84 (t, *J* = 7.4 Hz, 2H), 1.81–1.72 (m, 2H), 1.64 (m, 2H), 1.53 (m, 2H), 1.22 (m, 4H), 0.81 (m, 3H). ^13^C-NMR (DMSO-*d_6_*) δ 158.0, 155.9, 153.0, 149.3, 147.9, 143.9, 132.8, 130.5, 130.2, 130.1, 129.3, 129.2, 125.4, 124.1, 122.2, 119.2, 118.9, 117.1, 60.3, 31.8, 31.2, 30.8, 28.5, 25.4, 24.7, 21.6, 13.6. HRMS (ESI): calcd. for C_31_H_33_N_5_O_2_Na [M + Na]^+^; 530.2526; found: 530.2510.

*Methyl 4-{1-[6-(4-{[tert-butoxycarbonyl]amino}phenyl)-3-pentylcinnolin-4-yl]-1H-1,2,3-triazol-4-yl} benzoate* (**11e**). This compound was prepared in accordance with the general procedure from methyl 4-ethynylbenzoate **10e** (10.7 mg, 0.067 mmol), 4-azidocinnoline **7e** (29.0 mg, 0.067 mmol), CuSO_4_∙5H_2_O (0.84 mg, 0.0034 mmol), and sodium ascorbate (1.22 mg, 0.067 mmol) in a mixture of THF/H_2_O (3 mL); reaction time – 2 h. The crude product was purified by column chromatography (eluent: hexane/acetone = 3:1) to afford triazole **11e** as a yellow solid (27 mg, 68%). ^1^H-NMR (DMSO-*d_6_*) δ 9.56 (s, 1H), 9.40 (s, 1 H), 8.70 (d, *J* = 9.0 Hz, 1H), 8.36 (dd, *J* = 9.0, 1.9 Hz, 1H), 8.19–8.15 (m, 2H), 8.13–8.09 (m, 2H), 7.72–7.67 (m, 2H), 7.62–7.56 (m, 2H), 7.54 (d, *J* = 1.9 Hz, 1H), 3.89 (s, 3H), 2.97–2.93 (m, 2H), 1.77–1.64 (m, 2H), 1.47 (s, 9H), 1.26–1.15 (m, 4H), 0.80–0.72 (m, 3H). ^13^C-NMR (DMSO-*d_6_*) δ 165.9, 152.8, 152.6, 149.4, 146.2, 144.4, 140.5, 134.4, 131.1, 130.5, 130.0, 129.2, 128.7, 128.1, 126.0, 125.7, 122.0, 118.4, 116.3, 79.4, 52.2, 31.2, 30.7, 28.5, 28.0, 21.6, 13.6. HRMS (ESI): calcd. for C_34_H_36_N_6_O_4_Na [M + Na]^+^; 615.2690; found: 615.2683.

*4-{4-[4-(4-{Dimethylamino}phenyl)-1H-1,2,3-triazol-1-yl]-3-pentylcinnolin-6-yl}benzonitrile* (**11f**). This compound was prepared in accordance with the general procedure from 4-ethynyl-*N,N*-dimethylaniline **10f** (25.4 mg, 0.175 mmol), 4-azidocinnoline **7f** (60.0 mg, 0.175 mmol), CuSO_4_∙5H_2_O (2.19 mg, 0.0088 mmol), and sodium ascorbate (3.19 mg, 0.017 mmol) in the mixture of THF/H_2_O (6 mL); reaction time − 4 h. The crude product was purified by column chromatography (eluent: hexane/acetone = 3:1) to afford triazole **11f** as a yellow solid (74 mg, 86%). ^1^H-NMR (DMSO-*d_6_*) δ 9.04 (s, 1H), 8.79–8.76 (m, 1H), 8.41–8.37 (m, 1H), 7.96–7.95 (m, 4H), 7.85–7.75 (m, 2H), 7.69–7.68 (m, 1H), 6.84 (d, *J* = 8.4 Hz, 2H), 3.00 (t, *J* = 7.6 Hz, 2H), 2.96 (s, 6H), 1.82–1.57 (m, 2H), 1.23–1.17 (m, 4H), 0.86–0.57 (m, 3H). ^13^C-NMR (DMSO-*d_6_*) δ 153.1, 150.4, 149.5, 147.9, 142.8, 142.5, 133.1, 130.6, 129.2, 128.6, 126.5, 122.8, 121.7, 119.4, 118.5, 117.4, 112.3, 111.6, 31.3, 30.7, 28.5, 21.6, 13.6. HRMS (ESI): calcd. for C_30_H_30_N_7_ [M + H]^+^; 488.2557; found: 488.2558.

*4-(4-(3,4-Dimethoxyphenyl)-1H-1,2,3-triazol-1-yl)-3-pentyl-6-(thiophen-3-yl)cinnoline* (**11g**). This compound was prepared in accordance with the general procedure from 4-ethynyl-1,2-dimethoxybenzene **10b** (27.6 mg, 0.170 mmol), 4-azidocinnoline **7g** (55.0 mg, 0.170 mmol), CuSO_4_∙5H_2_O (2.12 mg, 0.0085 mmol), and sodium ascorbate (3.70 mg, 0.017 mmol) in the mixture of THF/H_2_O (6 mL); reaction time − 4 h. The crude product was purified by column chromatography (eluent: hexane/EtOAc = 2:1) to afford triazole **11g** as a yellow solid (68 mg, 82%). ^1^H-NMR (DMSO-d_6_) δ 9.17 (s, 1H), 8.68 (dd, *J* = 9.0, 0.6 Hz, 1H), 8.44 (dd, *J* = 9.0, 1.9 Hz, 1H), 8.23 (dd, *J* = 2.9, 1.4 Hz, 1H), 7.69 (dd, *J* = 5.1, 2.9 Hz, 1H),7.61–7.60 (m, 3H), 7.56 (dd, *J* = 8.2, 2.0 Hz, 1H), 7.11 (d, *J* = 8.4 Hz, 1H), 3.86 (s, 3H), 3.82 (s, 3H), 2.97–2.93 (m, 2H), 1.74–1.67 (m, 2H), 1.24–1.20 (m, 4H), 0.82–0.74 (m, 3H). ^13^C-NMR (DMSO-d_6_) δ 153.0, 149.4, 149.2, 149.1, 147.4, 139.4, 139.3, 130.4, 130.1, 128.9, 128.2, 126.4, 125.6, 124.0, 122.6, 122.3, 118.2, 116.2, 112.2, 109.3, 55.61, 55.60, 31.2, 30.8, 28.6, 21.6, 13.6. HRMS (ESI): calcd. for C_27_H_27_N_5_O_2_SNa [M + Na]^+^; 508.1778; found: 508.1790. Single crystals of **11g** were grown from the solution in chloroform by the vapor exchange with hexane. Crystal data for **11g** C_27_H_27_N_5_O_2_S (M = 485.59 g/mol): triclinic, space group P$\bar{1}$ (no. 2), a = 8.2511(5) Å, b = 10.9441(6) Å, c = 14.6090(6) Å, α = 105.898(5)°, β = 94.236(4)°, γ = 107.263(5)° V = 1194.17(12) Å3, Z = 2, T = 115(3) K, μ(CuKα) = 1.489 mm^−1^, D_calc_ = 1.350 g/cm^3^, 9946 reflections measured (6.382° ≤ 2Θ ≤ 139.978°), 4507 unique (Rint = 0.0378, Rsigma = 0.0505) which were used in all calculations. The final R_1_ was 0.0516 (I > 2σ(I)) and wR_2_ was 0.1510. Crystallographic data (excluding structure factors) for the structures reported in this work have been deposited with the Cambridge Crystallographic Data Centre as supplementary publication no. CCDC 1902675 (Supplementary Materials).

*3-Pentyl-6-(4-(trifluoromethyl)phenyl)cinnolin-4-amine* (**14**). To a stirred solution of 4-azidocinnolines **7b** (50.0 mg, 0.13 mmol) in DMSO (0.3 mL) at room temperature were propanal **12** (22.0 mg, 30.0 μL, 0.39 mmol) followed by 1,8-diazabicyclo[5.4.0]undec-7-ene (DBU) (6.0 mg, 5.8 μL, 0.039 mmol). The reaction mixture was stirred at room temperature for 3 hours (TLC control). The resulting mixture was diluted with water (10 mL) and extracted with ethyl acetate (3 × 8 mL). The combined organic layers were dried over anhydrous Na_2_SO_4_ and concentrated under reduced pressure to yield the crude product, which was purified by column chromatography on silica gel. ^1^H-NMR (acetone-*d*_6_) δ 8.60 (d, *J* = 1.9 Hz, 1H), 8.24 (d, *J* = 8.9 Hz, 1H), 8.08 (d, *J* = 8.7 Hz, 3H), 7.85 (d, *J* = 8.2 Hz, 2H), 6.51 (s, 2H), 3.23–3.04 (m, 2H), 1.93–1.79 (m, 2H), 1.49–1.35 (m, 4H), 0.90 (t, *J* = 7.0 Hz, 3H). ^13^C-NMR (acetone-*d*_6_) δ 149.0, 144.9 (^5^*J_C–F_* = 1.2 Hz), 143.4, 139.5, 138.7, 130.7, 130.1 (^2^*J_C–F_* = 32.3 Hz), 129.0, 128.9, 126.7 (^3^*J_C–F_* = 3.8 Hz), 125.4 (^1^*J_C–F_* = 271.2 Hz), 120.6, 116.1, 32.6, 32.3, 28.3, 23.3, 14.4.HRMS (ESI): calcd. for C_20_H_21_F_3_N_3_ [M + H]^+^; 360.1682; found: 360.1674.

*1,5-Ditosyl-7,8-didehydro-2,3,4,5,6,9-hexahydro-1H-1,5-diazonine* (**15**). To a stirred at 0 °C solution of Co_2_(CO)_6_-complex of **15** (100.0 mg, 0.139 mmol) [69] in acetone (3 mL) tetrabutylammonium fluoride hydrate (195 mg, 0.696 mmol, calculated for TBAF monohydrate) was added. The reaction mixture was allowed to warm to room temperature and then was stirred at this temperature for 1.5 hours (TLC control). After completion of the reaction the mixture was diluted with water (10 mL) and extracted with ethyl acetate (3 × 15 mL). The combined organic layers were dried over anhydrous Na_2_SO_4_ and concentrated under reduced pressure to yield the crude product, which was purified by column chromatography on silica gel (eluent: hexane/acetone = 3:1) to afford diazacyclononyne **15** as a white solid (56 mg, 93%). Spectral data were identical to those reported previously [69]. ^1^H-NMR (CDCl_3_) δ 7.69–7.62 (m, 4H), 7.37–7.30 (m, 4H), 3.79 (s, 4H), 3.31–3.24 (m, 4H), 2.44 (s, 6H), 2.14–2.09 (m, 2H).

*1-[3-Pentyl-6-(thiophen-3-yl)cinnolin-4-yl]-5,9-ditosyl-1,4,5,6,7,8,9,10-octahydro-*[1,2,3]*triazolo-[4,5-g]*[1,5]*- diazonine* (**16**). A mixture of diazacyclononyne **15** (58 mg, 0.133 mmol) and 4-azidocinnoline **7g** (43.0 mg, 0.133 mmol) in acetonitrile (2 mL) was stirred at 30 °C for 5 h. The solvent was removed under reduced pressure and the crude product was purified by column chromatography on silicagel usin hexane/acetone (3:1) as the eluent to give triazole **16** as a yellowish solid (95 mg, 95%). ^1^H-NMR (DMSO-*d_6_*) δ 8.71 (d, *J* = 9.0 Hz, 1H), 8.48 (dd, *J* = 9.0, 1.9 Hz, 1H), 8.22 (dd, *J* = 2.9, 1.4 Hz, 1H), 7.84 – 7.77 (m, 2H), 7.69 (dd, *J* = 5.2, 2.9 Hz, 1H), 7.59 (dd, *J* = 5.1, 1.4 Hz, 1H), 7.49 (d, *J* = 8.0 Hz, 2H), 7.32 (d, *J* = 2.0 Hz, 1H), 7.18–7.09 (m, 4H), 4.78 (d, *J* = 16.2 Hz, 1H), 4.62 (d, *J* = 16.3 Hz, 1H), 4.18 (s, 2H), 3.24 (dt, *J* = 7.3, 3.6 Hz, 2H), 3.22–3.11 (m, 1H), 2.98–2.90 (m, 1H), 2.68–2.61 (m, 1H), 2.43 (s, 3H), 2.27 (s, 3H), 1.91–1.74 (m, 4H), 1.30–1.22 (m, 4H), 0.86–0.78 (m, 3H). ^13^C-NMR (DMSO-*d_6_*) δ 154.2, 149.3, 143.8, 143.7, 143.2, 139.6, 139.2, 134.6, 134.5, 133.9, 130.4, 130.3, 130.0, 129.7, 128.2, 127.3, 126.8, 126.6, 126.2, 125.6, 122.8, 115.6, 48.7, 48.6, 45.3, 42.1, 31.0, 31.0, 29.4, 28.5, 21.7, 21.0, 20.8, 13.7. HRMS (ESI): calcd. for C_38_H_42_N_7_O_4_S_3_ [M + H]^+^: 756.2455; found: 756.2466.

### 3.3. DFT Calculations

All computations were carried out at the DFT/HF hybrid level of theory using Becke’s three-parameter hybrid exchange functional in combination with the gradient-corrected correlation functional of Lee, Yang, and Parr (B3LYP) by using the GAUSSIAN 2003 program packages [76]. The geometries optimization was performed using the 6-311+G(2d,2p) basis set (standard 6–311 basis set added with polarization (d, p) and diffuse functions). Optimizations were performed on all degrees of freedom, and optimized structures were verified as true minima with no imaginary frequencies. The Hessian matrix was calculated analytically for the optimized structures in order to prove the location of correct minima and to estimate the thermodynamic parameters.

### 3.4. The Absolute Fluorescence Quantum Yield Measurements

The absolute fluorescence quantum yield was measured on a Horiba Fluorolog-3 spectrometer (Edison, NJ, USA) equipped with an integrating sphere. A xenon lamp coupled to a double monochromator was used as excitation light source. The sample (1 cm quartz cuvette cell with molecular solution in THF) or blank (pure THF) were directly illuminated in the center of the integrating sphere. The optical density of all investigated sample solutions in THF did not exceed 0.1 at the luminescence excitation wavelength. Under the same conditions (e.g., excitation wavelength, spectral resolution, temperature), the luminescence spectrum of the sample *E_c_*, the luminescence spectrum of the blank *E_a_*, the Rayleigh scattering spectrum of the sample *L_c_*, and the Rayleigh scattering spectrum of the solvent *L_a_* were measured. The absolute fluorescence quantum yield was determined according to the formula:

(1) $$\Phi =\left(E_{c}-E_{a}\right)/\left(L_{a}-L_{c}\right)$$

### 3.5. Cell Culture Cultivation and Cytotoxicity Studies

The HeLa cells were obtained from the Russian Collection of Cell cultures (Institute of Cytology RAS, Saint Petersburg, Russia). Cells were cultivated in DMEM medium containing 10% FBS (fetal bovine serum) at 37 °C and 5% CO_2_. Compounds were diluted with DMSO to concentrations of 7.5; 5; 2.5; 0.5 and 0.1 mM. These solutions were diluted 100 x times with culture medium to the final concentrations of 100, 75, 50, 25, 5 and 1 μM/L (1% DMSO). Cytotoxicity assays were made using 3-(4,5-dimethyl-2-thiazolyl)-2,5-diphenyl-2*H*-tetrazolium bromide (MTT) [77]. Cells were placed in 96-well plates (5000 of cells per well in 100 μL of DMEM + FBS without compounds) and preincubated for 24 hours. Subsequently culture medium was aspirated and 100 μL volumes of media with compounds were added to the wells. After 24 h of drug exposure, cytotoxicity assays were performed using MTT. Twenty μL of MTT solution (5 mg/mL) was added to the cell cultures. During the following 3 h of incubation mitochondrial dehydrogenase activity of vital cells caused the production of formazan crystals from MTT. Culture medium was aspirated and formazan was dissolved in 100 μL of DMSO. The optical density at 570 nm, which is directly proportional to the number of surviving cells, was measured using microplate reader Uniplan AIFR-01 (Pikon, Moscow, Russia).

## 4. Conclusions

In summary, a convenient synthetic route towards 4-azido-6-arylcinnolines based on Suzuki coupling, Richter-type cyclization and nucleophilic substitution of activated aromatic bromine atom with azido function was developed. The crucial point in the synthetic sequences is to carry out the Suzuki coupling as the initial step and the introduction of an azido group as the final step. Despite the possibility of chemoselective substitution of the bromine atom at C4 in 4,6-dibromocinnoline with the formation of 4-azido-6-bromocinnoline, the azide moiety at C4 of cinnoline ring was found to be unstable under Suzuki reaction conditions. The synthetic route developed here is functional group tolerant to various substituents on the aryl moiety. All 4-azidocinnolines underwent CuAAC with terminal alkynes providing 4-triazolylcinnolines in moderate to high yields. The reactivity of 4-azidocinnolines in SPAAC was also confirmed. Whereas 4-azidocinnolines display fluorescent properties under excitation at their low wavelength absorption maximum, the corresponding 4-triazolylcinnolines were devoid of any fluorescence, probably due to their non-planar geometry and PET mechanism. Among the series of 4-triazolylcinnolines synthesized the compound **11a** bearing a hydroxyalkyl-substituted triazole ring was found to be cytotoxic toward HeLa cells at micromolar concentrations.

## Figures and Tables

**Table 1:** Absolute QY for THF solutions of 6-aryl-4-azifocinnolines **7a−g**.

Entry	Azidocinnoline7 (R)	Quantum yield, %
1	**7a** (H)	0.8
2	**7b** (CF_3_)	0.1
3	**7c** (OMe)	0.9
4	**7d** (OPh)	0.3
5	**7e** (NHBoc)	0.8
6	**7f** (CN)	0.2
7	**7g** (3-thieny) ^1^	0.0

^1^ Attached directly to C6 of cinnoline core.

**Table 2:** Optimized geometries of azidocinnolines, triazolylcinnolines and their frontier molecular orbitals.

Entry	Compound ^1^	Torsion Angle ^2^	HOMO (E, ev)	LUMO (E, ev)
1	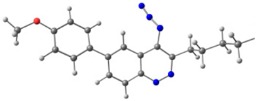 **7c**	**−**	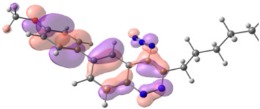 **−6.23**	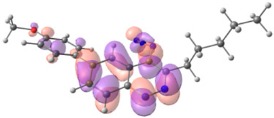 **−2.43**
2	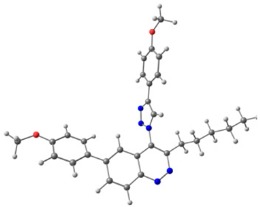 **11c**	106.3°	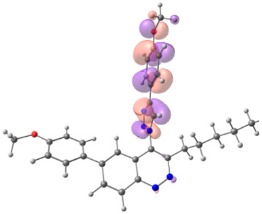 **−5.93**	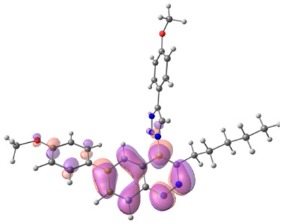 **−2.48**
3	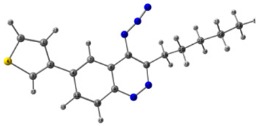 **7f**	**−**	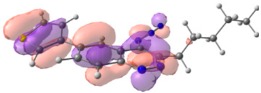 **−6.41**	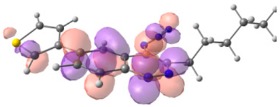 **−2.45**
4	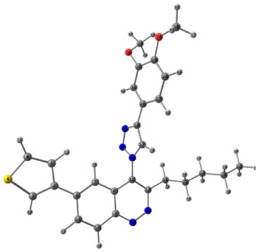 **11f**	100.0°	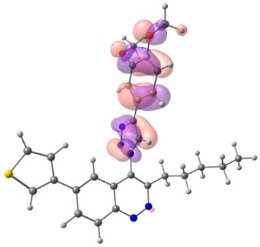 **−5.95**	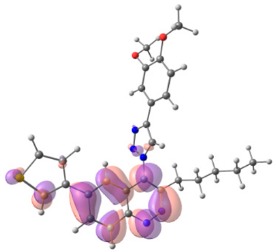 **−2.58**

^1^ Geometry optimization (B3LYP 6–311 + G(2d,2p)); ^2^ Torsion angle between triazole and cinnoline rings in optimized strucures.

**Figure A1:**
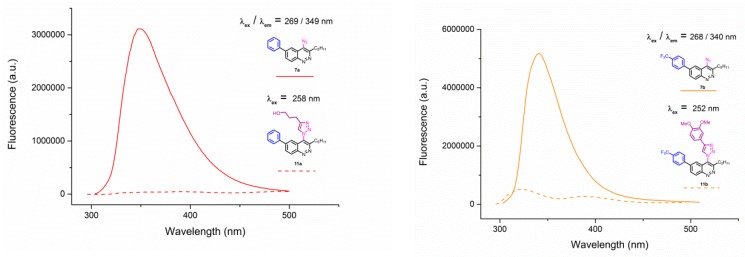
Emission spectra of 4-azidocinnolines/4-triazolylcinnolnes **7a**/**11a**; **7b**/**11b**; **7e**/**11e**; **7f**/**11f.**

## Supplementary Materials

The following are available online, copies of ^1^H, ^13^C{^1^H}, and DEPT NMR spectra for all newly synthesized compounds; Cartesian coordinates and energies and isosurfaces of MO for optimized geometries of **7c,f** and **11c,f**; cif file with X-ray data for compound **11g**.
